# 3,3′-Diindolylmethane (DIM): A Molecular Scaffold for Inhibition of WWP1 and WWP2, Members of the NEDD4 Family HECT E3 Ligases

**DOI:** 10.1021/acsomega.4c09944

**Published:** 2025-02-10

**Authors:** Ashley P. Dudey, Gregory R. Hughes, Jake M. Rigby, Serena Monaco, G. Richard Stephenson, Thomas E. Storr, Jesus Angulo, Andrew Chantry, Andrew M. Hemmings

**Affiliations:** † School of Biological Sciences, University of East Anglia, Norwich NR4 7TJ, United Kingdom; ‡ School of Chemistry, Pharmacy and Pharmacology, University of East Anglia, Norwich NR4 7TJ, United Kingdom; § Instituto de Investigaciones Químicas (CSIC-Universidad de Sevilla), Sevilla 41092, Spain; ∥ International Research Center for Food and Health, College of Food Science and Technology, Shanghai Ocean University, Nanhui New City, Shanghai 201306, P. R. China

## Abstract

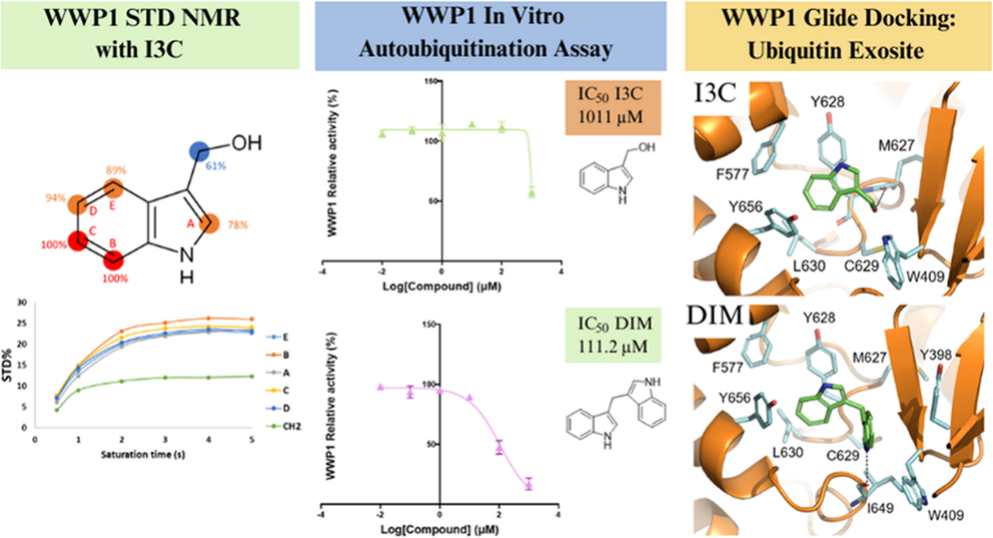

Indole-3-carbinol (I3C) is a metabolic derivative of glucobrassicin found in cruciferous vegetables. Known for its anticarcinogenic properties, I3C has been shown to target the NEDD4 family HECT E3 ligases, NEDD4-1 and WWP1, yet *in vitro* confirmation for the latter is lacking. Here, we characterize the interactions of I3C and a set of 17 derivatives with WWP1 and its homologue, WWP2. Saturation transfer difference (STD) NMR analysis confirmed strong interaction of I3C with WWP1 but weaker with WWP2. However, while autoubiquitination activity assays revealed weak inhibition of WWP1, the I3C condensation product, 3,3′-diindolylmethane (DIM), was more potent (IC_50_ 111.2 μM; 95% CI = 85.1, 145.8). Molecular modeling of DIM to the ubiquitin exosite of both enzymes suggested the WW2 domain makes hydrophobic interactions with the ligand that may contribute to inhibitory action. Taken together, our results suggest future drug lead development should focus on the SAR between WWP1 and DIM.

## Introduction

All eukaryotes employ the ubiquitin (Ub) system to post-translationally modify substrate proteins with the 76 amino acid protein, ubiquitin, thus targeting them for degradation and other outcomes. The Ub system operates as a catalytic cascade involving three key enzymes: Ub-activating (E1), Ub-conjugating (E2), and Ub-ligating (E3). These enzymes work together to attach Ub to specific proteins, resulting in various forms of mono- or poly ubiquitination.^1^ Dysregulation of this system has been linked to several diseases, particularly cancer, which has spurred efforts to discover new therapeutic options.^2^

E3 ligases present an appealing target for drug discovery since they determine the substrate specificity of the Ub system, potentially allowing for targeted intervention in specific cancer pathways.^3^ There are over 600 E3 ligases, classified into three subtypes based on their Ub transfer mechanisms: Really Interesting New Gene (RING), Homologous to E6AP Carboxyl Terminus (HECT), and RING Between RING (RBR).^4−6^ HECT E3 ligases are particularly promising for drug development as they participate directly in Ub transfer, relying on a single active site cysteine for their catalytic activity. Several HECT E3 ligases have been implicated in tumor initiation and progression, with the NEDD4 family drawing the most attention due to their significant role in malignancies. Among the NEDD4 family, WW domain-containing E3 ligases 1 (WWP1) and 2 (WWP2) are of particular interest as they target the tumor suppressor protein phosphatase and tensin homologue (PTEN) and other tumor suppressors and transcription factors.^7,8^ Dysregulation of WWP1 and WWP2 has been directly associated with cancer, as well as various neurological, inflammatory, and infectious diseases, including COVID-19.^9−12^ Currently, only a limited number of small-molecule inhibitors for HECT E3 ligases have been identified, all with low micromolar potency. Only two inhibitors have been reported to specifically target WWP1: the commercially available HECT ligase inhibitor Heclin, with an IC50 of 6.9 μM, and indole-3-carbinol (I3C) and its derivatives, which have shown potential in cell proliferation studies.^7,13^

**Figure 1:**
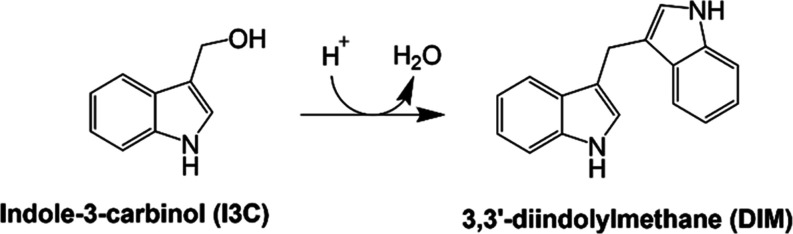
Acid catalyzed conversion of I3C to DIM.

Cruciferous vegetables have long been associated with health benefits, from compounds with high nutritional value to bioactive phytochemicals.^14^ A significant proportion of the pro-health phytochemicals have been largely accredited to the enzymatic breakdown of glucosinolates,^15^ in particular glucobrassicin, known to produce the metabolic product, I3C.^16^ I3C has been extensively studied for its broad therapeutic potential, which has been shown to display antitumor, antioxidative, antiviral, and antimicrobial properties.^17^ However, these diverse properties may also be attributed to the acid-catalyzed condensation products of I3C, mainly 3,3′-diindolylmethane (DIM), rapidly produced in acidic environments such as those found in the stomach (Figure 1).^18^ To no surprise, both I3C and DIM can be purchased as health supplements and are under various clinical trials, mainly associated with their effects on breast and prostate cancer.^19,20^

Current investigations into I3C-protein interactions have identified three targets. Inhibition of the serine protease elastase was the first to be discovered and found to result in the disruption of NF-kB signaling causing cell cycle arrest and apoptosis in breast cancer cell lines.^21−23^ I3C has more recently been found to target two members of the NEDD4 family of HECT E3 ligases, NEDD4-1 (neural precursor cell expressed developmentally down-regulated protein 4) and WWP1 (WW domain containing E3 ubiquitin protein ligase 1). These studies have not only discovered an anticarcinogenic characteristic of I3C through interfering with NEDD4-1/WWP1 mediated ubiquitination of tumor suppressor protein PTEN (phosphatase and tensin homologue) but also have highlighted an antiviral property shown to prevent viral budding in COVID-19.^24−26^

With NEDD4-1 and WWP1 already known to be promising therapeutic targets, various studies have looked to characterize their interactions with I3C, aiming to increase both its acid stability and potency for future lead development. It has been suggested that I3C binds to a ubiquitin (Ub) exosite and likely prevents ubiquitin chain elongation.^27^ This proposal was based on the observation of an I3C-derived covalent inhibitor found bound to the noncatalytic Cys627 of NEDD4-1 (PDB ID: 5C91).^28^ This site has since been utilized to computationally dock I3C to develop various more potent and stable inhibitors, including 1-benzyl-I3C shown to decrease the IC_50_ from 284 to 12.3 μM. This derivative was also found to be highly potent in MCF-7 breast cancer cell growth studies when compared to I3C.^29^ However, this is likely a result of a dual action as 1-benzyl-I3C has also been shown to target elastase with increased potency over I3C.^23^ The suggestion of a I3C-targeting Ub exosite in WWP1 is supported by site mutations in the proposed binding pocket resulting in both an increased *K*_D_ and loss of I3C sensitivity in cell proliferation assays.^25^ Interestingly, this study also demonstrated that cells with WWP1 deletions over NEDD4-1 were more resistant to I3C suggesting WWP1 to be the direct target. Thermal shift analysis has also been used to confirm I3C interactions with the HECT domain, resulting a 3.8 °C shift in *T*_m_.^30^ Despite this, in vitro inhibition of WWP1 by I3C, its bioactive condensation product, DIM, or other stable derivatives has not been determined.

Here, we look to characterize interactions between I3C and WWP1 using the more sensitive saturation transfer difference (STD) NMR technique to confirm binding. Subsequently, we used an in vitro autoubiquitination assay to measure inhibition by I3C as well as a range of its derivatives, either purchased or synthesized, including literature derived acid-stabilized analogues. Given its high amino acid sequence homology in the HECT domain and proposed binding site, we further expanded this approach to WWP2 (WW domain containing E3 ubiquitin protein ligase 2), a 70% sequence homologue of WWP1 and therapeutic target. Finally, molecular docking was utilized to help understand the structure–activity relationship (SAR) of I3C with these enzyme targets.

## Results and Discussion

**Figure 2:**
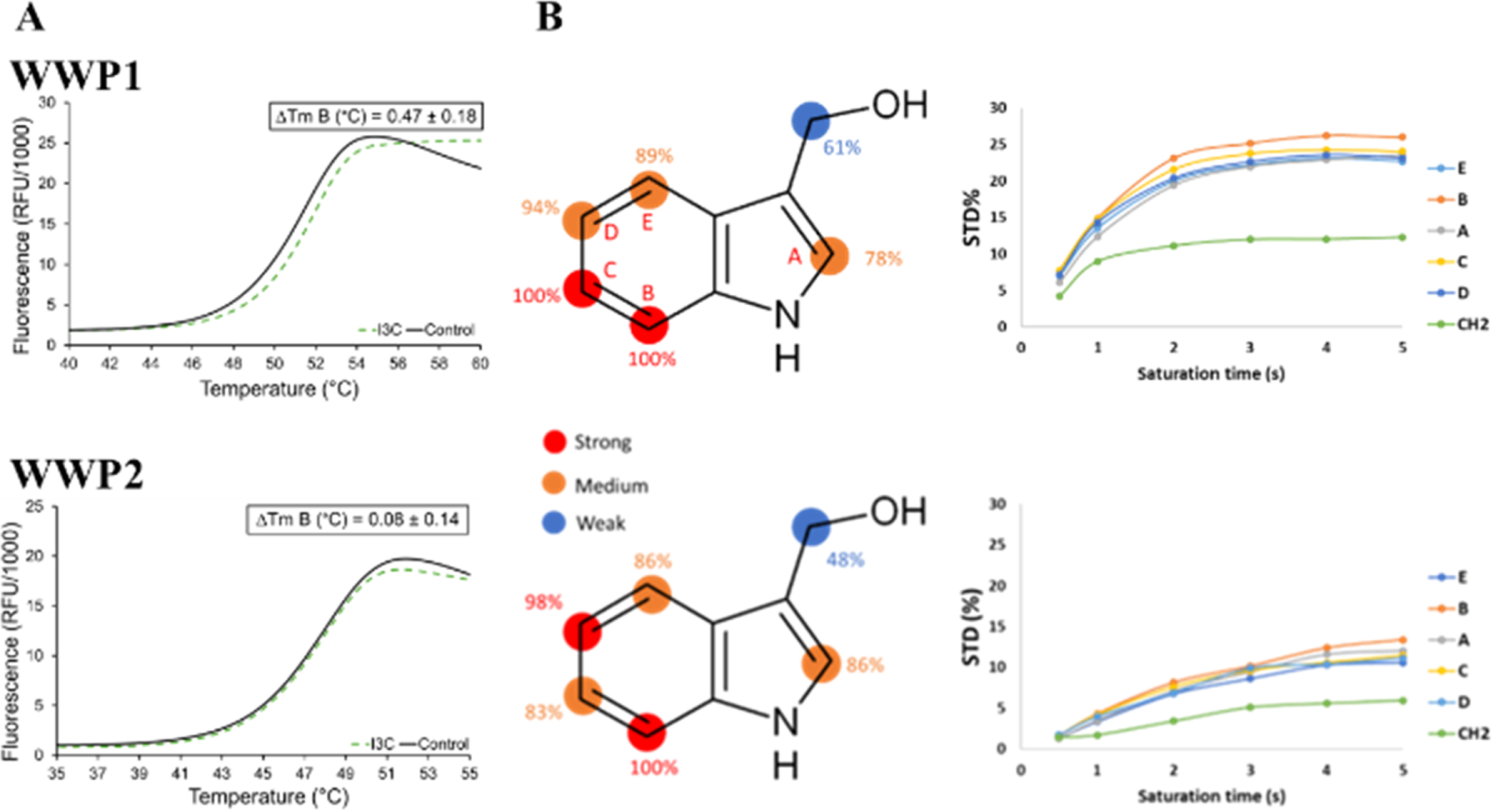
Binding of indole-3-carbinol (I3C) to WWP1 (top) and WWP2 (bottom). (A) DSF melt curve in the presence (green dashed) and absence (black line) of I3C. Triplicate assays were carried out using 100 μM I3C, containing 0.1% dimethyl sulfoxide (DMSO) with Tm B calculated using the standard Boltzmann fit. (B) STD NMR binding epitope maps (selective protein irradiation at 0.0 ppm) of I3C based on the normalized saturation transfer intensities (0–100%, left). Binding epitope maps were obtained from the initial slope of the build-up curves for each proton (right). Legend indicates weak (blue), medium (orange) and strong (red) intensities. Raw STD spectra are reported in SI Figure S1.

Differential scanning fluorimetry (DSF) was utilized to initially assess interactions of I3C with both WWP1 and WWP2. Although binding of I3C to WWP1 has been reported previously,^25^ we wanted to determine whether such interactions are observed with a protein construct containing the WW2 domain, given its proximity to the proposed exo binding site. In this regard, the WWP1-WW2-2,3-linker-WW3-WW4-HECT (WWP1-2L34H) and WWP2-WW2-2,3-linker-HECT (WWP2-LH) constructs were chosen (SI Figure S2) and DSF assays performed using 100 μM I3C (0.1% DMSO) with midpoint melting temperature (*T*_m_) determined by fitting a Boltzmann regression to the melting curve (Figure 2A). Three independent repeat experiments were performed. WWP1 was found to exhibit an average Δ*T*_m_ beyond three times the standard deviation of the negative controls, albeit with a modest shift (0.47 ± 0.15 °C Δ*T*_m_). This is in marked contrast to that seen when using the simple WWP1 HECT domain as a target.^30^ WWP2 displayed no significant shift (0.07 ± 0.11 °C Δ*T*_m_).

Given the similarities between WWP1 and WWP2 HECT domains, we speculated that the relatively low sensitivity of DSF may not be able to detect weak WWP2-I3C interactions and therefore opted to investigate further using STD NMR. STD NMR is a versatile ligand-based NMR approach, relying on selective saturation of the protein. This saturation is transferred to the ligand when and if the ligand binds, causing a reduction of the signal intensities. The STD intensities are calculated as the difference of the reference spectra (where selective saturation is off) minus the irradiated spectra, and they are proportional to the vicinity of the protein surface. This feature allows us to obtain the binding epitope mapping, i.e., a map of the ligand protons which are in closer contact to the protein, determining the ligand binding mode. To gain structural insights into I3C binding to the two different enzymes, we used the STD NMR build-up curves approach to obtain the binding epitope map of the two complexes (Figure 2B). The STD NMR binding experiments confirmed that I3C binds to both WWP1 and WWP2. For the WWP1-I3C complex, the binding epitope map is in very good agreement with the in silico model proposed in literature,^25^ having the C-D-E-F ring buried more deeply in the binding cleft, and the carbinol moiety being more solvent exposed. The strong STD intensities observed, approaching 30%, are also in agreement with the strong binding affinity (*K*_D_ 450 nM) found in the literature.^25^ By comparison, the STD intensities observed for the WWP2-I3C complex were much lower, compatible with a lower binding affinity. The binding epitope map is also harder to interpret, as protons *in para* to each other (B and D, and C and E) have comparable STD intensities: B and D show very strong intensities while C and E show medium intensities. This could suggest that the ligand is interacting in multiple binding modes or at different sites.

**Figure 3:**
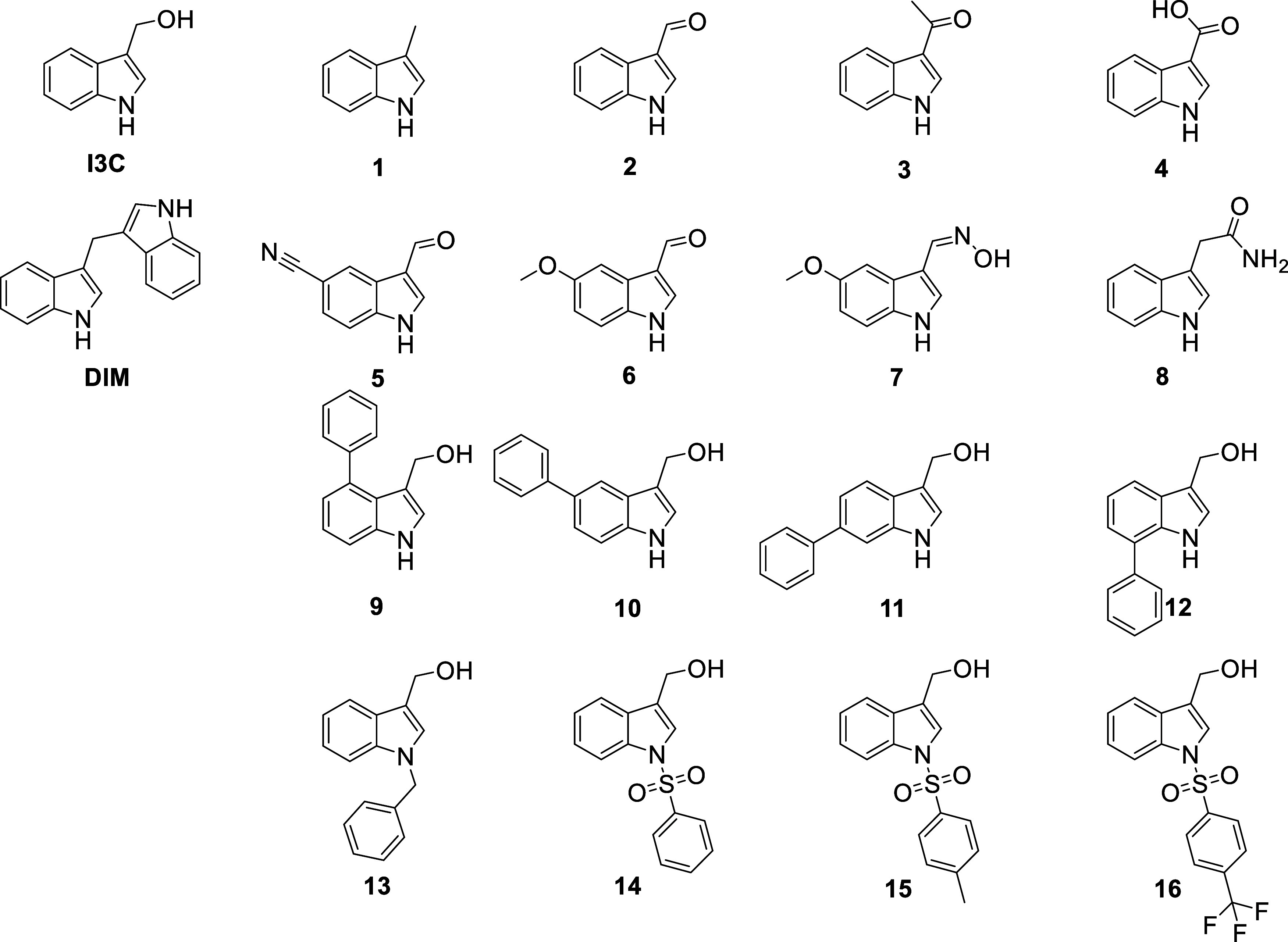
Chemical structures of I3C, DIM and I3C derivatives (labeled 1–16) with associated numbering.

To explore the chemical space of I3C interactions with WWP1 and WWP2, we generated a series of derivatives (Figure 3). These included both those commercially available as well as more stable synthesized analogues found in literature to target WWP1 and NEDD4-1.^29−31^ This necessitated the synthesis of DIM and compounds **13**, **15** and **16**, and full details are presented in the SI.

A single shot *in vitro* autoubiquitination assay was performed for I3C, DIM and each of the derivatives against WWP1 and WWP2 using a compound concentration of 1 mM (1% DMSO) (SI Figure S3). The purpose of this assay was to rapidly identify those compounds which demonstrated significant inhibition. These active compounds were then subjected to a detailed dose dependency analysis leading to IC_50_ values. This strategy has previously been employed to screen other WWP1/WWP2 small molecule inhibitors as well as used to assess I3C inhibition toward NEDD4-1.^29,32^ In this way, compounds displaying a relative activity (RA) of less than 50% in the single shot assay were further screened for dose-dependency using a logarithmic concentration range from 10 nM to 1 mM (1% DMSO) (SI Figure S4). Notably, we did not experience difficulties in terms of the solubility of any of the compounds tested at 1 mM concentration in 1% DMSO. Only a small subset of analogues (I3C, DIM, 15 and 16) displayed inhibition of WWP1, and only compounds 15 and 16 displayed inhibition of WWP2. The results are summarized in Table 1. These were confirmed by counter assays to remove compounds possibly interacting with other enzymes in the assay or interfering with the assay directly (SI Table S1).

**Table 1:**
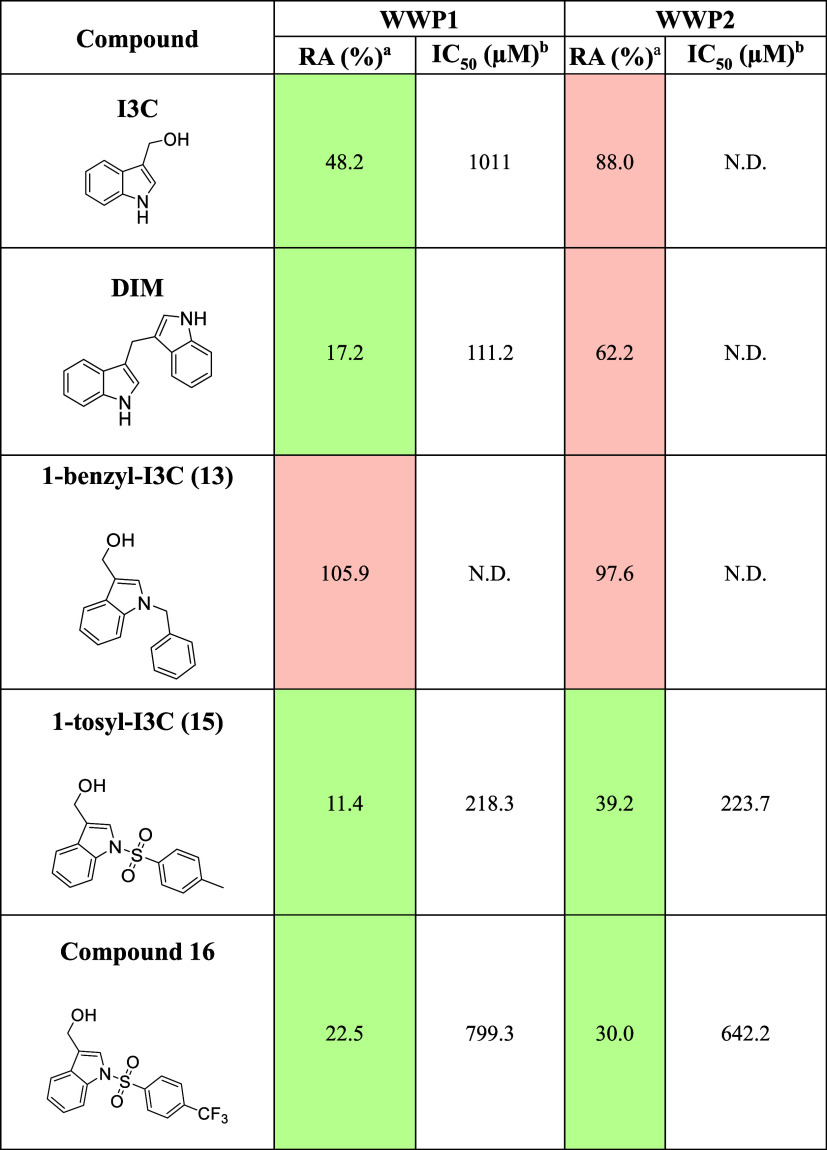
Summary of Results of Analysis of I3C Derivatives by Autoubiquitination Assay

a Relative activity (RA) standardized to 0 and 100% controls, compound at 1 mM (1% DMSO). Only compounds showing an RA < 50% (cells colored green) were subject to autoubiquitination assay. For details of the standardization procedure employed see the autoubiquitination assay Experimental Section of the SI.

b IC_50_ values calculated from nonlinear regression of RA against log concentration (1 mM to 10 nM, at 1% DMSO) standardized to 0 and 100% controls. ND, not determined.

I3C displayed only weak inhibition toward WWP1 in the single shot assay (48.2 ± 8.6% RA) and no significant inhibition of WWP2 was observed (88.0 ± 7.5% RA). Despite this we carried out a full dose dependence assay for inhibition of WWP1 and were able to derive an estimated IC_50_ of 1011 μM. However, numerical instability led to failure of calculation of an associated confidence interval. Attempts to derive a more accurate estimate failed due to issues with solubility of the compound at higher concentrations. This result may be compared with a dissociation constant of 450 nM previously determined by microscale thermophoresis for interaction of I3 with the WWP1 HECT domain.^25^ Although *K*_D_ and IC_50_ values cannot be compared directly, we presume that the marked difference in these results stems at least in part from the nature of the enzyme construct adopted for the analysis (i.e., HECT domain vs WWP1-L34H). Our result also contrasts with the reported inhibition of NEDD4-1 by I3C (IC_50_ 284 μM) and runs counter to the suggestion that I3C targets WWP1 directly.^29^ Interestingly, the more stable condensation product DIM was more potent as an inhibitor toward WWP1 with an IC_50_ of 111.2 μM (95% CI = 85.1, 145.8). Inhibition by DIM was also observed toward WWP2, although substantially weaker (62.2 ± 6.1% RA). Given the poor stability of I3C in both acidic and to a lesser extent neutral conditions, its antiproliferation properties at least through WWP1 may indeed be a result of its conversion to the more potent DIM.^18,33^ Therapeutic trials of ‘I3C′ supplements demonstrated the conversion to DIM, having a maintained presence in the tissues studied.^34^ Interestingly, DIM has also been shown to target the Akt-PTEN signaling pathway, itself associated with WWP1 and WWP2 malignant properties.^35−37^

Surprisingly, compound **13** (1-benzyl-I3C) provided no evidence of inhibition of WWP1 or WWP2 (Table 1), despite being highlighted as a potent inhibitor of NEDD4-1.^29^ This demonstrates a possible degree of selectivity toward the HECT E3 ligases and is most likely a result of various point mutations between NEDD4-1 and WWP1/WWP2 in the proposed binding site. This includes the NEDD4-1 noncatalytic cysteine residue (Cys627 mutation to Ile649/Ile597), which is found instead on an adjacent stretch of polypeptide chain (Gly606 mutation to Cys629/Cys577).

Compounds **15** (1-tosyl-I3C) and **16** displayed inhibition toward WWP1 and WWP2, both containing a stabilizing moiety as either *N*-(4-methylphenyl)sulfonyl I3C or *N*-(4-trifluoromethylphenyl)sulfonyl I3C, respectively. 1-Tosyl-I3C was a more potent inhibitor with an IC_50_ of 218.3 μM (95% CI = 182.8, 261.8) toward WWP1 and 223.7 μM (95% CI = 130.0, 400.8) toward WWP2. These compounds were based on OSU-A9, an I3C analogue primarily designed to overcome I3C acid instability and shown to demonstrate a 100-fold increase in its antiproliferative properties.^31^

**Figure 4:**
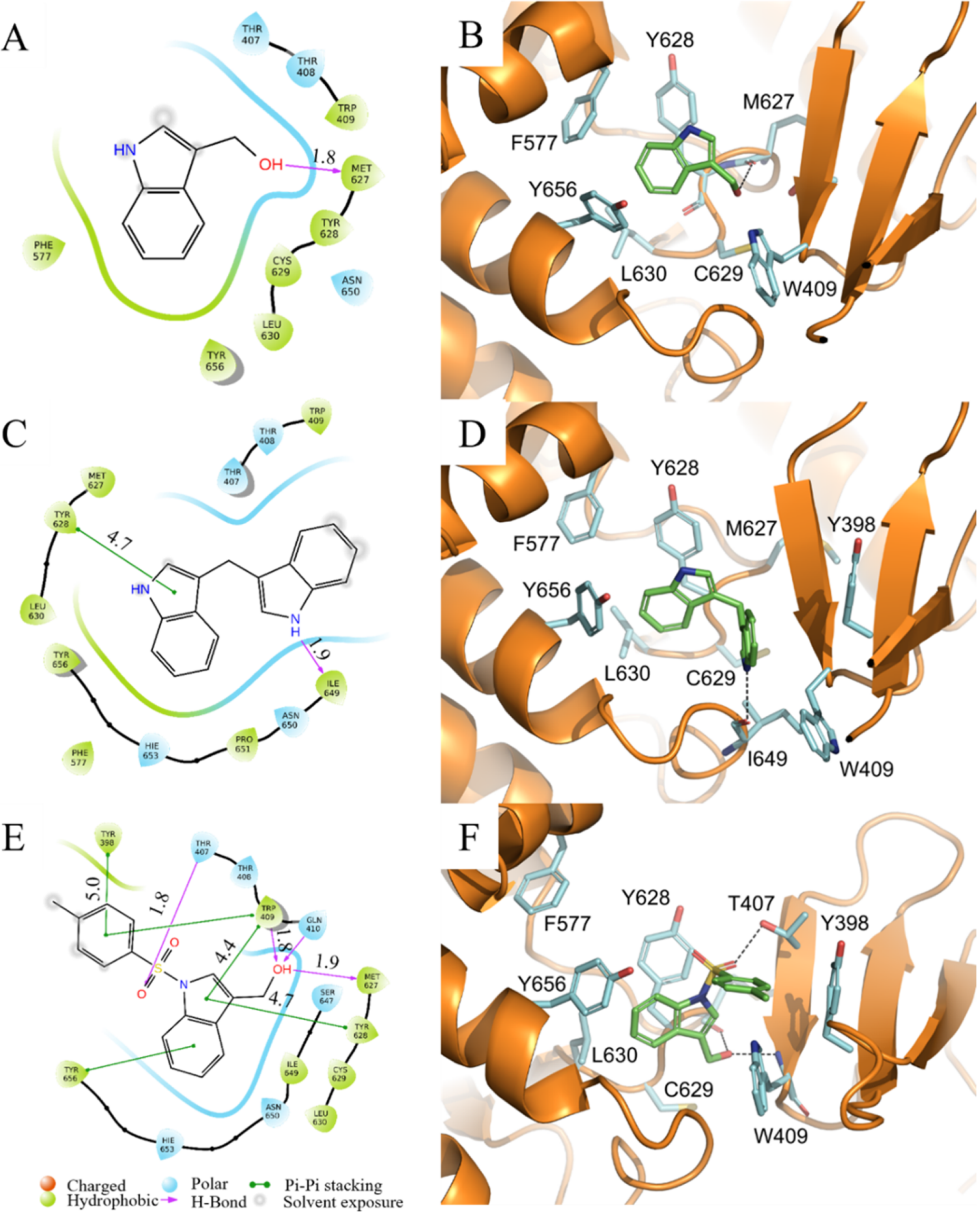
Ligand poses of I3C, DIM and 1-tosyl-I3C with WWP1. Compounds were minimized and redocked into the Ub exosite of PDB entry 9EQK using Glide software (Schrödinger Inc.). Panels (A), (C) and (E) show two-dimensional (2D) interaction interface diagrams taken from Schrodinger for I3C, DIM and 1-tosyl-I3C, respectively. Hydrophobic (green) and hydrophilic (blue) residues are shown with key polar (magenta arrow) and π–π (green arrow) interactions indicated and corresponding distances given in Ångstrom, Å. Panels (B), (D) and (F) show three-dimensional (3D) interaction interfaces for I3C, DIM and 1-tosyl-I3C, respectively. Key WWP1 residues are represented as sticks and labeled with carbon atoms colored cyan, nitrogen blue, oxygen red and sulfur yellow. Hydrogen bonds are shown as black dashed lines. To help distinguish the ligands their carbon atoms are colored green. All hydrogen atoms have been removed from the views. Images were taken from similar viewpoints and created in PyMOL.

To gain a better understanding of the SAR of I3C against WWP1 and WWP2, we explored the proposed binding site with I3C, DIM and 1-tosyl-I3C using in silico molecular docking. I3C has previously been modeled against the HECT domains of both NEDD4-1 and WWP1, resulting in the observation of interactions of the indole ring in a hydrophobic cavity located close to the noncatalytic cysteines.^25,29^ However, to also consider possible binding contributions of the WW2 domain, we superposed WWP1 (PDB ID: 9EQK) and WWP2 (PDB ID: 6J1Z) crystal structures containing the WW2 domain onto the previously solved crystal structure of NEDD4-1 with a covalently bound I3C-derivative inhibitor (PDB ID: 5C91), before aligning our stable I3C derivatives.^28^ With the WWP1 and WWP2 apo structures having much tighter binding pockets than bound NEDD4-1, we first minimized the surrounding residues to generate a pseudobound state, before using the Schrödinger Glide docking protocol to simulate ligand interactions. The resulting representative poses of I3C, DIM and 1-tosyl-I3C to WWP1 are shown in Figure 4, with WWP2 poses shown in SI Figure S5. The GlideScores for both WWP1 and WWP2 are found in SI Table S2.

GlideScores for I3C were the poorest of the compounds tested (−6.28 and −6.45 kcal/mol for WWP1 and WWP2, respectively), in agreement with weaker binding suggested by single shot assays. The second pose of I3C in complex with WWP1 was chosen due to its agreement with the STD NMR binding epitope (Figure 2B top). This pose places the indole group of the compound into a hydrophobic cavity formed from the side chains of WWP1 residues Phe577, Tyr628, Leu630 and Tyr656. Interestingly, residue Phe577 is mutated to leucine (Leu553) in NEDD4-1, presumably leading to a larger cavity. A single hydrogen bond is predicted between the I3C hydroxyl group and the mainchain carbonyl oxygen of Met627. Although having a marginally better GlideScore, WWP2-I3C generated a range of dissimilar poses of similar energies suggesting a weaker overall preference for binding which aligned well with the results from STD NMR analysis (Figure 2B bottom).

Docking of DIM produced more favorable GlideScores (−7.09 and −7.79 kcal/mol for WWP1 and WWP2, respectively), following the trend in RA values seen in single shot assays (Table 1). DIM makes similar contacts with both WWP1/WWP2 to those seen with I3C: not only does the indole group bind in the hydrophobic pocket but it also makes π–π stacking interactions with Tyr628/Tyr576. It also features hydrogen bonds with the polypeptide backbone of Ile649/Ile597, the residue equivalents of Cys627 in NEDD4-1. DIM makes hydrophobic interactions with Tyr398/Tyr347 and Trp409/Trp358 residues located on the WW2 domain (Figure 4D). Compared with I3C, the additional steric bulk of the second indole group of DIM displaces residue Trp409 of WWP1 forcing it to rotate approximately 180° around the side chain torsion angle, γ1, thereby resulting in an increase in volume of the exosite. This suggests a plasticity of the binding site which may prove a fruitful avenue for exploitation in future studies.

1-tosyl-I3C (**15**) displayed the most favorable GlideScores, −8.44 and −7.28 kcal/mol for WWP1 and WWP2, respectively, with poses predicted to make a variety of contacts with residues of both the HECT and WW2 domains. Although positioned somewhat further out of the hydrophobic cavity than seen for I3C and DIM, the indole group still participates in π–π stacking with Tyr656/Tyr604 and the hydroxyl group forms hydrogen bonds to the Tyr628/Tyr576 and Trp409/Trp358 backbones. The 1-tosyl moiety makes closer contacts to Tyr398/Tyr347 and Trp409/Trp358 enabling π–π stacking, with the sulfonyl oxygen forming a hydrogen bond to Thr407/Thr357 also located on the WW2 domain. Although possessing a greater steric bulk than I3C, the cumulative effect of these interactions is a pose which does not result in expansion of the binding pocket by reorientation of residue W409/Trp358 as is predicted for DIM.

At this stage, it is difficult to distinguish the inhibitory action of DIM and 1-tosyl-I3C beyond blocking the Ub exosite. This task was made more complex given that our autoinhibitory assays exploited a variation of WWP1 lacking the C2 and WW2 domains. This choice of enzyme construct was dictated by the strong autoinhibition mechanism observed in full-length WWP1 constructs making them mostly inactive.^38^ However, although this demonstrates that the WW2 interactions are not vital for the inhibition of these I3C derivatives, they may still contribute to their inhibitory action. The WW2 domain is known to be involved in an autoinhibitory mechanism, also shown to block the Ub exosite, and aid in positioning the 2,3-linker region to prevent C-lobe movement, a vital aspect of HECT activity.^39^ Interestingly such interactions do not occur in NEDD4-1 itself, shown to bind C2 in this region through an alternative mechanism.^24^ Differences in I3C, DIM and 1-tosyl-I3C inhibition discussed herein alongside residue substitutions in this pocket suggest significant differences in the binding environment and therefore the possibility of a selective therapeutic approach between NEDD4-1 and WWP1/WWP2.

## Conclusions

In conclusion, the bioactive metabolite I3C was confirmed to bind to WWP1 via the proposed Ub exosite supported through STD NMR epitope maps and in silico molecular docking. I3C was also indicated to interact with WWP2 although more weakly than with WWP1. Despite this, I3C itself displayed minimal inhibition, with the most potent derivative of those tested being its acid condensation product, DIM. 1-tosyl-I3C, another acid-stabilized derivative based on the antiproliferative analogue OSU-A9 also displayed modest potency in the mid-μM range against both WWP1 and WWP2.^31^ Molecular docking of DIM and 1-tosyl-I3C to WWP1 and WWP2 suggests the WW2 domain makes hydrophobic, π–π and hydrogen bonding interactions with the ligands and may contribute to their inhibitory action through strengthening an autoinhibitory state.^38^ Interestingly, the NEDD4-1 inhibitor 1-benzyl-I3C showed no evidence of interaction with either WWP1 or WWP2, and alongside significant differences between the proposed binding sites may support the generation of a class of selective HECT NEDD4 family inhibitors. With various DIM derivatives already shown in the literature to display increased antiproliferative properties, future studies should consider targeting WWP1 in a guided SAR approach to develop a lead compound for cancer therapeutics.^19^

## Materials and Methods

An expanded explanation of the methodologies employed, including further details of the chemical, biological, spectroscopic and computational methods is available in the Supporting Information.

### Chemical Materials

Unless specified, all reagents and starting materials were purchased from commercial sources (Sigma-Aldrich (Merck Life Sciences), Fluorochem (Doug Discovery), Fischer Scientific, Alfa Aesar) and used as supplied. Indole-3-carbinol was purchased from Fluorochem and used as received (97% purity). Thin-layer chromatography was performed on Merck silica gel 60 F254 plates and visualized by UV absorption, purchased from VWR International. Flash column chromatography was carried out using Silica Gel 60 purchased from Material Harvest. “Concentrated” refers to the removal of volatile organic solvents via distillation using a rotary evaporator. “Dried” refers to pouring onto or adding anhydrous MgSO_4_ or Na_2_SO_4_ to (as specified), followed by filtration. Water refers to deionized water.

### Chemical Methods

Details of the syntheses of DIM and compounds **13**, **15**, and **16** can be found in the Additional Chemical Experimental Section of the Supporting Information. NMR spectra were recorded on 400 or 500 MHz Bruker NMR spectrometer using the deuterated solvent stated in the reported data. ^1^H, ^13^C and 19F NMR samples were prepared by dissolving a sample in 0.4–0.7 mL deuterated solvent. All deuterated solvents were purchased from Cambridge Isotopes and used as received, solvents were stored under 4 Å molecular sieves after opening. All spectra were referenced to the residual solvent peaks of the solvent used.2 NMR spectra chemical shifts (δ) are reported in ppm and coupling constants (*J*) are reported in hertz (Hz). Abbreviations for NMR splitting are s (singlet), d (doublet), t (triplet), q (quartet), and m (multiplet). Infrared spectra were recorded using a PerkinElmer Spectrum Two LITA. High-resolution mass spectrometry was performed at the University of East Anglia using a UPLC-HRMS (ACQUITY H-Class PLUS UPLC and Waters SYNAPT XS High Resolution Mass Spectrometer) setup with electrospray ionization using ca. 1 μg/mL solution in acetonitrile or methanol. Melting points (not corrected) were recorded on a Büchi Melting Point B-545 using capillary melting point tubes made in-house. Compounds **7**, **9**, **10**, **11**, **12**, **13**, **14**, **15**, **16** and DIM were synthesized and are all 95–99% purity as determined by ^1^H NMR analysis. Oxime **7** existed as an approximately 1:1 mixture of cis and trans isomers.

### Biological Materials

All reagents were purchased from ThermoFisher, Sigma-Aldrich and Melford, unless otherwise stated. All plasmids were either purchased from Addgene or kindly gifted (Table S3).

### Biological Methods

Further details of biological methods can be found in the Supporting Information.

### DNA Techniques

Plasmids (Table S3) were transformed using standard heat shock or electroporation, incubating for 12–18 h at 37 °C on LB agar plates containing respective antibiotics (Table S4).

### Protein Expression and Purification Techniques

All protocols were performed on ice or at 4 °C unless otherwise stated. Transformed *Escherichia coli* cells were inoculated and incubated overnight at 37 °C, 180 rpm in LB containing appropriate antibiotics. The desired recombinant proteins were expressed, by induction with IPTG at OD_600_ 0.6–1.0 before incubating at protein specific conditions (Table S4). Cells were pelleted by centrifugation (Beckman Coulter J20, JLA 8.1000 rotor) at 4000*g*, 4 °C for 30 min and stored at −20 °C. Cells were lysed by either using a 4710 series ultrasonic homogenizer CP50 (Cole-Parmer) at 50% amp for 10 s on, 10 s off for a total of 6 min or French pressed at 16,000 psi using a precooled pressure cell (Thermo French Press). Affinity columns (Cytiva Life Sciences) were installed onto a benchtop peristaltic pump (Parnachia Biotech) at room temperature or AKTA Pure 2 system (Cytiva Life Sciences) at 4 °C following supplier protocols. Sample concentration was achieved using 5 or 10 kDa MW cut off Vivaspin protein concentrators (GE Healthcare), centrifuged (Beckman Coulter J-15R, JS-4.750 rotor) at 4000*g* for 10 to 20 min per spin with mixing. All samples were snap-frozen and stored at −80 °C unless otherwise stated. Details of the procedures followed, and the buffers used to purify the individual ligases used in this work are available in the Additional Biological Experimental Section of the Supporting Information.

### Differential Scanning Fluorimetry

A 96 well-plate (MicroAmp Optical) was loaded with 18 μL of 3.8 μM WWP1-2L34H and 2.5 μM WWP2-LH, in their respective buffers (SI Table S5) containing 5 × SYPRO orange dye. A 2 μL aliquot of the compound was added to a final concentration of 100 μM containing 0.1% DMSO before the plate was sealed (MicroAmp Clear Adhesive Film). Both nonprotein and DMSO controls were also generated. The plates were briefly centrifuged before the assay was run using an ABI 7500 RT-PCR following the melt curve using ROX (575 nm) as the “preset” fluorescence dye. A standard thermal profile of 25–70 °C, rising at 0.5 °C per minute was used. The midpoint melting temperature (*T*_m_) was calculated using a Boltzmann fit to the fluorescence curve using Protein Thermal Shift Software v1.4 (ThermoFisher). Results were further processed and plotted using Excel.

### Autoubiquitination Assay

Cell lysate containing His-tagged WWP1-L34H or GST-tagged WWP2-FL proteins were incubated on either 96-well Clear Pierce glutathione or nickel-coated plates for 1 h. Reaction mixtures of either 3 ng/well GST-Uba1 and 15 ng/well UbcH7 or 10 ng/well His-Uba1 and 150 ng/well His-UbcH7 were incubated together in 25 mM Tris pH 8.0, 100 mM NaCl, 4 mM MgCl_2_ containing 60 ng/well FLAG-ubiquitin and 1.25 mM ATP for 40 min. A prior 1% BSA plate blocking step is required for nickel-coated plates. After plate washing, 2 μL of the compound was added at the desired concentration (1% DMSO) followed by 18 μL of the reaction mixture. This was then incubated for 2 h with 0 and 100% controls before 100 μL of anti-FLAG M2-Peroxidase HRP (1:10,000 PBST) was added to each well and incubated for 1 h. Finally, 100 μL of 1 × TMB substrate solution (Invitrogen) was added to each well and incubated for up to 10 min until sufficient blue color change was observed. To stop the reaction, 100 μL of 1 M HCl was added. For the counter assay, 3 ng/well GST-Uba1 and 200 ng/well UbcH7 were incubated with the other reaction mixture components for 1 h before incubating onto plates for a further 1 h. All other steps were followed. The plates were washed three times with PBST (and 15 mM Imidazole) between each step. Quantification was measured by absorbance read at 450 nm. All assay optimizations were as previously reported.^32^ IC_50_ nonlinear regression curves were calculated in GraphPad v10.2 (Prism).

### Saturation Transfer Difference (STD) NMR

An Amicon centrifuge filter unit with a 10 kDa MW cutoff was used to exchange the protein in 25 mM d_19_-2,2-bis(hydroxymethyl)-2,2′,2″-nitrilotriethanol, 100 mM NaCl and 1.0 mM DTT buffer pH* 8.9 (uncorrected for the deuterium isotope effect on the pH glass electrode) in D_2_O. The STD NMR sample was composed of 500 μM indol-3-carbinol and 20 μM protein (WWP1 and WWP2, respectively). For all STD NMR experiments, the on- and off-resonance spectra were acquired using a train of 50 ms Gaussian selective saturation pulses using a variable saturation time, with on-resonance frequency at 0.0 ppm and off-resonance frequency at 40 ppm. The binding epitope mapping determination (STD build-up curves) was obtained at incremental saturation times from 0.5 to 5 s. Residual protein resonances were filtered out using a *T*_2_ filter of 40 ms. All the STD NMR experiments were performed with a spectral width of 10 kHz and 32768 data points using 256 or 512 scans. All the NMR experiments were performed on a Bruker Avance 800.23 MHz at 278 K. Binding epitope mappings were obtained by determining the initial slopes (STD_0_) calculated by performing a least-squares fitting of the following monoexponential curve:
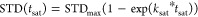
where STD(*t*_sat_) is the STD intensity for a saturation time, *t*_sat_, STD_max_ is the maximum STD intensity and *k*_sat_ is the rate constant for saturation transfer. In the limit, *t*_sat_ →:
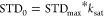
Importantly, STD_0_ gives a value that is independent of any relaxation or rebinding effects, allowing for a more accurate binding epitope. The value of STD_0_ was then normalized against the proton with the largest intensity to give values in the range of 0–100%, which were then mapped onto the ligand structure to give the corresponding binding epitope mapping.

### Molecular Docking

Molecular docking was performed using the Schrödinger Suite 2020-3. The protein structures of NEDD4 HECT (PDB ID: 5C91),^28^ as well as WWP1 (PDB ID: 9EQK) and WWP2 (PDB ID: 6J1Z)^38^ both containing the HECT and the WW2 domains, were prepared using the Schrödinger’s Protein Preparation Wizard module (Epik v5.5, Impact v8.8).^40,41^ I3C, DIM, as well as 1-tosyl-I3C and compound 16, were prepared using LigPrep v5.5 (Epik v5.3).^42^ Default settings were used for both proteins and ligands at pH 7.0 ± 0.2, removing all waters and adding hydrogen atoms. Both WWP1 and WWP2 structures and ligands were aligned to NEDD4 and its covalent I3C analogue, before performing minimization to both the ligand and residues surrounding an 8 Å radius. This was achieved using the OPLSe force field in MacroModel v12.9 at a default 2500 iterations.^40,41^ The ligands were then redocked into the minimized pseudobound structures using the Glide SP v8.8 program with grids generated from the individual minimized ligand positions.^43,44^ Default settings were used with the top five poses generated, enabling postligand minimization before being ranked and binding affinity given as GlideScore. Figures were created in 2D using the Schrodinger Ligand Interaction Diagram module, with 3D molecular models generated using PyMOL v2.5^45^

## Supporting Information Available

Details of the results of chemical compound characterization, as well as details of the synthetic, analytical, biological and computational procedures used (PDF)

## Abbreviations

C2: Ca^2+^ binding domain
DIM: 3,3′-diindolylmethane
DMSO: dimethyl sulfoxide
DSF: differential scanning fluorimetry
HECT: homologous to the E6-AP carboxyl terminus
I3C: indole-3-carbinol
MCF-7: Michigan cancer foundation-7
NEDD4: neural precursor cell expressed developmentally down-regulated protein 4
PTEN: phosphatase and tensin homologue
RA: relative activity
SAR: structure–activity relationship
SI: supporting Information
STD-NMR: saturation transfer difference-nuclear magnetic resonance spectroscopy
WW: tryptophan–tryptophan domain
WWP1: WW domain-containing E3 ubiquitin protein ligase 1
WWP1-2L34H: WWP1-WW2-2,3-linker-WW3-WW4-HECT
WWP2: WW domain-containing E3 ubiquitin protein ligase 2
WWP2-LH: WWP2-WW2-2,3-linker-HECT
