# Supplementary material for: 3,3′-Diindolylmethane (DIM): A Molecular Scaffold for Inhibition of WWP1 and WWP2, Members of the NEDD4 Family HECT E3 Ligases
Source: ACS Omega. 2025 Feb 10;10(6):5963–72. doi: 10.1021/acsomega.4c09944 (PMC11840788; doi:10.1021/acsomega.4c09944)
Supplement: Supplementary file 1 — ao4c09944_si_001.pdf [file ao4c09944_si_001.pdf]

# SUPPORTING INFORMATION

# 3,3′-diindolylmethane (DIM): A Molecular Scaffold for Inhibition of WWP1 and WWP2, Members of the NEDD4 Family HECT E3 Ligases.

Ashley P. Dudey[a], Gregory R. Hughes[a], Jake M. Rigby[b], Serena Monaco[b], G. Richard Stephenson[b], Thomas E. Storr [b], Jesus Angulo[b,c], Andrew Chantry [a], and Andrew M. Hemmings[a,b,d]*

[a]*School of Biological Sciences, University of East Anglia, Norwich NR4 7TJ, United Kingdom*

[b]*School of Chemistry, Pharmacy and Pharmacology, University of East Anglia, Norwich NR4 7TJ, United Kingdom*

[c]*Instituto de Investigaciones Químicas (CSIC-Universidad de Sevilla), 41092, Sevilla, Spain*

[d]*International Research Center for Food and Health, College of Food Science and Technology, Shanghai Ocean University, Nanhui New City, Shanghai, P.R. China. Zip Code:201306.*

*Corresponding author

**Andrew M. Hemmings**
School of Biological Sciences, University of East Anglia, Norwich Research Park, Norwich NR4 7TJ. UK. and International Research Center for Food and Health, College of Food Science and Technology, Shanghai Ocean University, Shanghai 201308.
Email: andrew@shou.edu.cn; a.hemmings@uea.ac.uk

# Contents

**Supporting Figures for Results and Discussion ........................................................................S5**

**Additional Biological Experimental Section..........................................................................S11**

Materials.........................................................................................................................S11

DNA Techniques..............................................................................................................S11

Protein Purification Techniques......................................................................................S11

Protein Expression ......................................................................................................S12

Protein Purification .....................................................................................................S12

SDS-PAGE Analysis.........................................................................................................S17

Differential Scanning Fluorimetry (DSF) .......................................................................S22

Auto-ubiquitination Assay ..............................................................................................S22

Saturation Transfer Difference (STD) NMR ......................................................................23

Molecular Docking .........................................................................................................S25

**Additional Chemical Experimental Section ..........................................................................S26**

General procedure 1: PTC sulfonamide formation ........................................................S28

General procedure 2: N-(aryl)indole-3-carboxaldehyde reduction.................................S29

Compound Synthesis........................................................................................................S30

**Compound 13A –** N-benzylindole-3-carboxaldehyde ...................................................S30

**Compound 15A** – N-(4-methylbenzenesulfonyl)indole-3-carboxaldehyde .....................S32

**Compound 16A** – N-(4-trifluoromethylbenzenesuflonyl)indole-3-carboxaldehyde........S34

**Compound 13** – N-benzylindole-3-carbinol ..................................................................S37

**Compound 15** – N-(4-methylbenzenesulfonyl)indole-3-carbinol ...................................S39

**Compound 16** – N-(4-trifluoromethylbenzenesulfonyl)indole-3-carbinol .......................S41

**DIM** – 3,3'-diindolylmethane.........................................................................................S44

**Additional References...........................................................................................................S46**

## List of Tables

**Table S1.** Counter auto-ubiquitination screen of Hit I3C derivatives. (p.S9)

**Table S2.** Glide scoring of I3C, DIM and 1-tosyl-I3C against WWP1 and WWP2. (p.S9)

**Table S3.** Plasmids, constructs, and their origins. (p.S11)

**Table S4.** *E. coli* cell-line and protein expression conditions. (p.S12)

**Table S5.** Proteins and their respective buffers. (p.S16)

## List of Figures

**Figure S1**. STD NMR binding experiments for indol-3-carbinol (I3C) in complex with WWP1 and WWP2, obtained at 2 s saturation time, with selective protein irradiation at 0.0 ppm. (p.S5)

**Figure S2**. Schematic diagram of the WWP1 and WWP2 construct architecture. (p.S6)

**Figure S3.** Single-shot Autoubiquitination screen of I3C derivatives against WWP1 (top) and WWP2 (bottom). (p.S7)

**Figure S4.** Dose-dependent auto-ubiquitination assay of single-shot hit I3C derivatives against WWP1 (top) and WWP2 (bottom). (p.S8)

**Figure S5.** Ligand poses of I3C, DIM and 1-tosyl-I3C with WWP2. Compounds were minimised and re-docked into the exo Ub site of PDB entry 6J1Z using Glide software. (p.S10)

**Figure S6.** SDS-PAGE analysis of WWP1-L34H purification. (p.S18)

**Figure S7.** SDS-PAGE analysis of WWP1-2L34H purification. (A) Gradient IMAC isolation. (p.S19)

**Figure S8.** SDS-PAGE analysis of WWP2-LH purification. (p.S19)

**Figure S9.** SDS-PAGE analysis of WWP1-HECT and WWP2-HECT purification. (p.S20)

**Figure S10.** SDS-PAGE analysis of Uba1 and UbcH7 purifications. (p.S21)

**Figure S11.** NMR data for **Compound 13A** – N-benzylindole-3-carboxaldehyde (p.S28)

**Figure S12.** NMR data for **Compound 15A** – N-(4-methylbenzenesulfonyl)indole-3-carboxaldehyde (p.S30)

**Figure S13.** NMR data for **Compound 16A** – N-(4-trifluoromethylbenzenesuflonyl)indole-3-carboxaldehyde (p.S35)

**Figure S14.** HRMS data for **Compound 16A** – N-(4-trifluoromethylbenzenesuflonyl)indole-3-carboxaldehyde (p.S36)

**Figure S15.** NMR data for **Compound 13** – N-benzylindole-3-carbinol (p.S38)

**Figure S16.** NMR data for **Compound 15** – N-(4-methylbenzenesulfonyl)indole-3-carbinol (p.S40)

**Figure S17.** NMR data for **Compound 16** – N-(4-trifluoromethylbenzenesulfonyl)indole-3-carbinol (p.S42)

**Figure S18.** HRMS data for **Compound 16** – N-(4-trifluoromethylbenzenesulfonyl)indole-3-carbinol (p.S43)

**Figure S19.** NMR data for **DIM** – 3,3'-diindolylmethane (p.S45)

## Supporting Figures for Results and Discussion

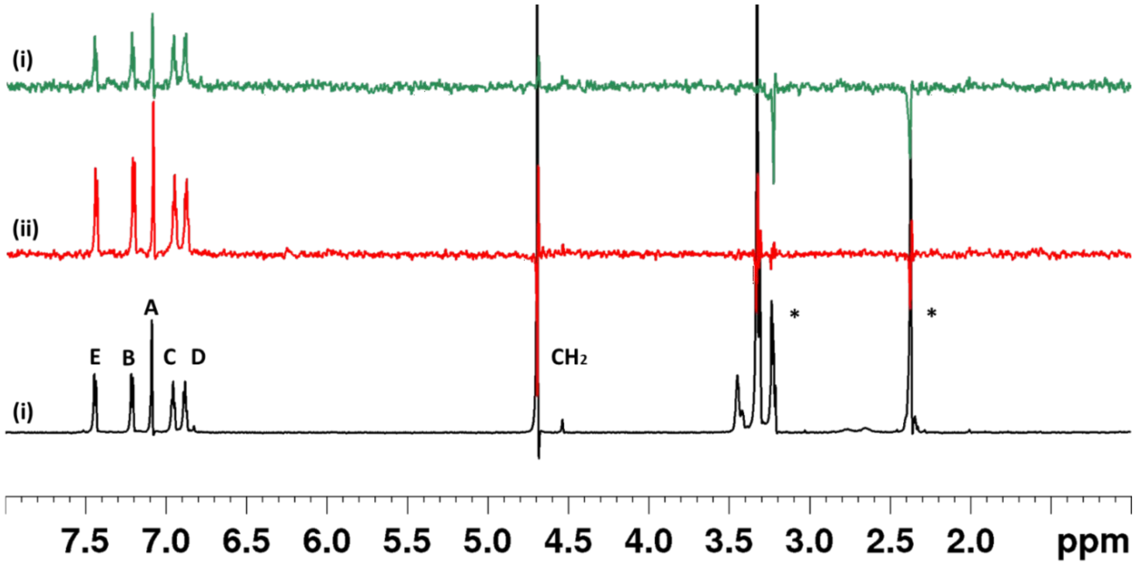


**Figure S1**. STD NMR binding experiments for indol-3-carbinol (I3C) in complex with WWP1 and WWP2, obtained at 2 s saturation time, with selective protein irradiation at 0.0 ppm. (i) Reference spectrum for the WWP1-I3C complex. ii) STD difference spectrum for the I3C-WWP1 complex. iii) STD difference spectrum for the WWP1-I3C. The ligand assignment is given as labels on the reference spectrum, while the residual buffer and d[6]-DMSO are labelled with a *.

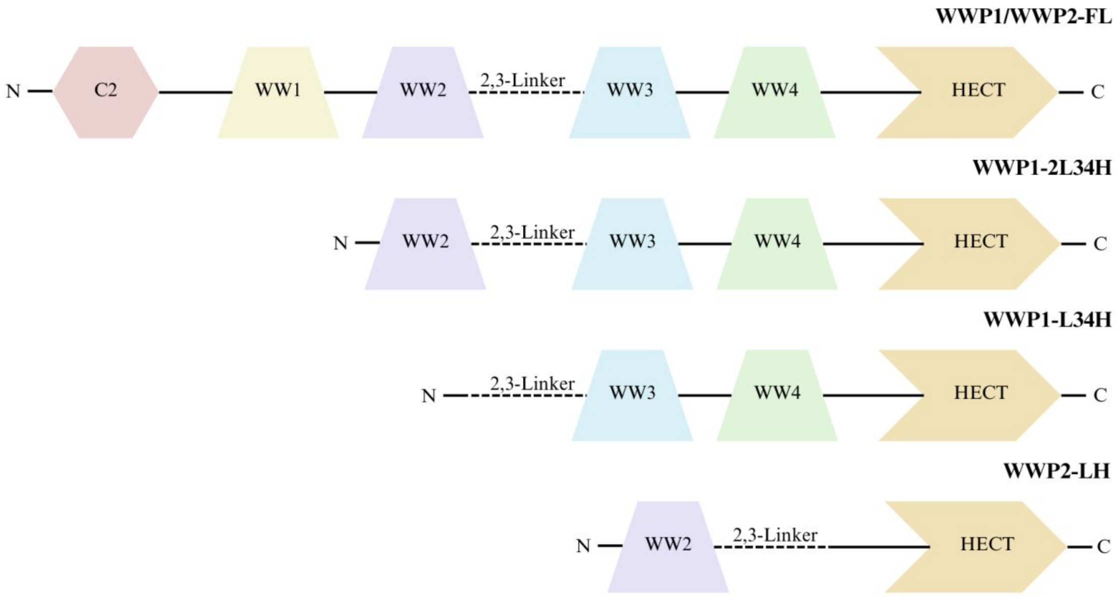


**Figure S2**. Schematic diagram of the WWP1 and WWP2 construct architecture. Full-length WWP2 (WWP2-FL), WWP1-WW2-2,3-linker-WW3-WW4-HECT (WWP1-2L34H), WWP1- 2,3-linker-WW3-WW4-HECT (WWP1-L34H) and WWP2-WW2-2,3-linker-HECT (WWP2-LH) are all shown, containing various arrangements of the $Ca^{2+}$ binding (C2) (pink), four Tryptophan-Tryptophan (yellow, purple, blue and green), and active site HECT (orange) domain alongside the 2,3-linker (dashed line) region vital for autoinhibition.

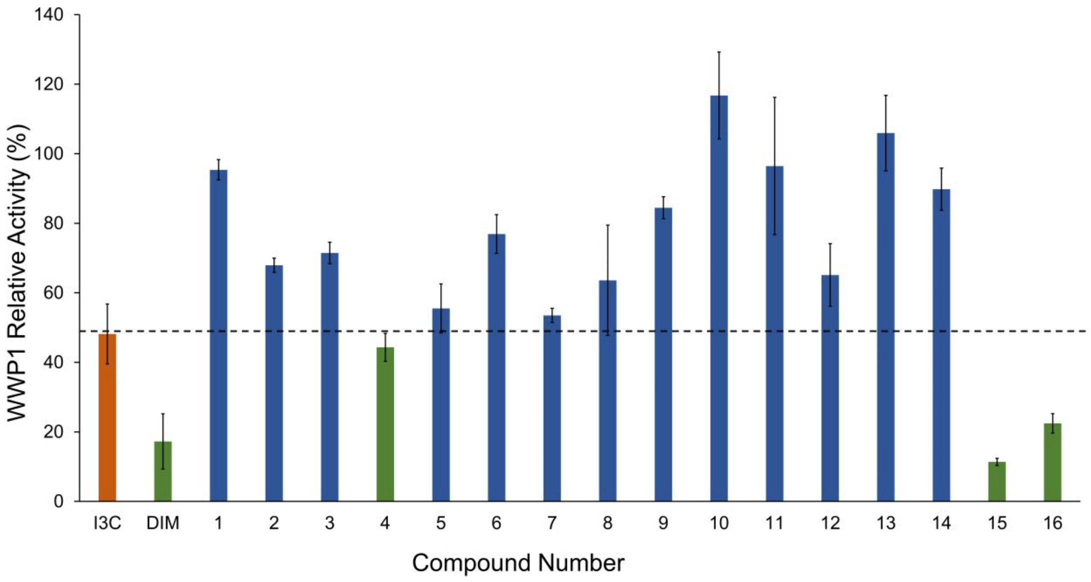


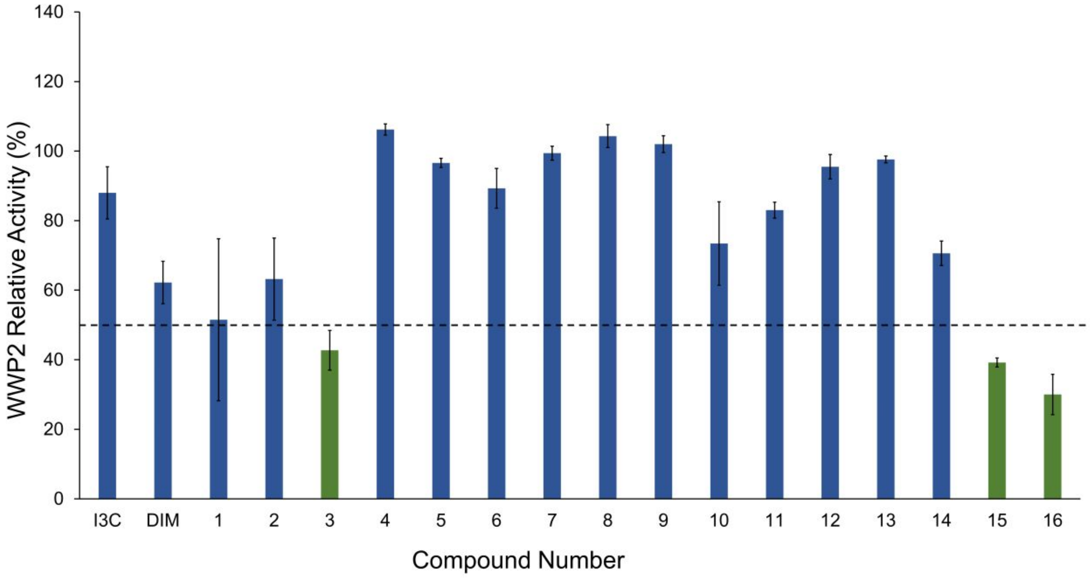


**Figure S3.** Single-shot Autoubiquitination screen of I3C derivatives against WWP1 (top) and WWP2 (bottom). Compound inhibition measured at 1 mM (1 % DMSO), with a hit threshold (green) of less than 50 % relative activity (dashed line), normalised to their respective 0 % and 100 % WWP1-L34H and WWP2-FL controls.

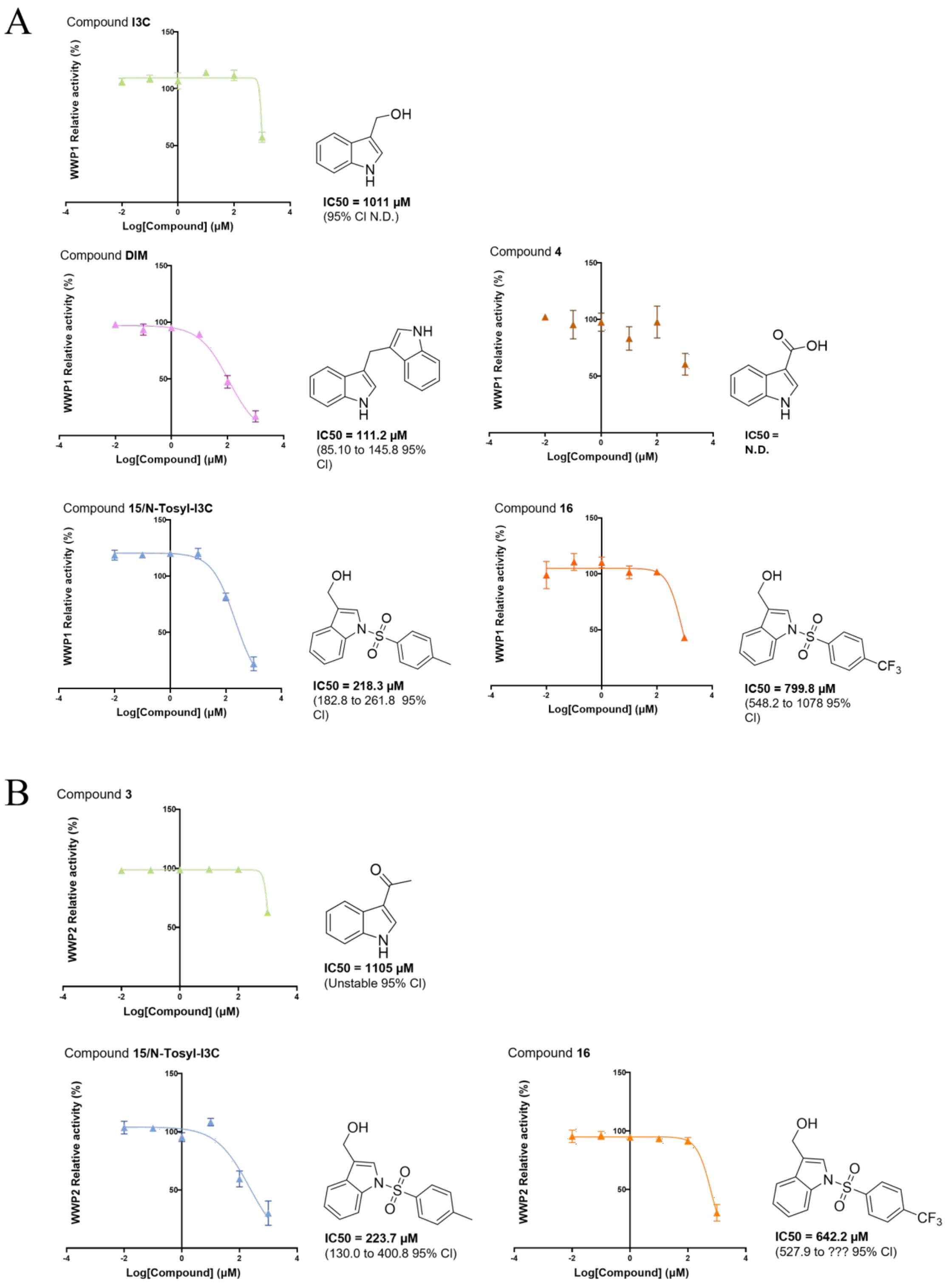


**Figure S4.** Dose-dependent auto-ubiquitination assay of I3C and derivatives against (A) WWP1 and (B) WWP2 . Compound inhibition measured on a log scale from 1 mM – 10 nM (1 % DMSO), normalised to 0 % and 100 % controls. $IC_{50}$ values calculated from non-linear regression curves fitted in GraphPad software. The 95 % confidence interval

is given in brackets. Note that it was not possible to determine the 95% confidence interval for the IC50 of I3C inhibition of WWP1.

**Table S1.** Counter auto-ubiquitination screen of hit I3C derivatives.

| Compound | UbcH7 Activity (%) |
|---|---|
| **DIM** | 83.3 ± 2.0 |
| **1-tosyl-I3C** | 86.3 ± 1.8 |
| **16** | 79.7 ± 1.5 |

**Table S2.** Glide scoring of I3C, DIM and 1-tosyl-I3C against WWP1 and WWP2.

| Compound | Structure | WWP1 | WWP2 |
|---|---|---|---|
| | | Glide Score (kcal/mol) | Glide Score (kcal/mol) |
| **I3C** | 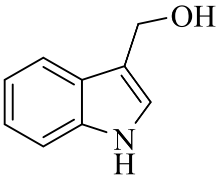 | - 6.28 | -6.45 |
| **DIM** | 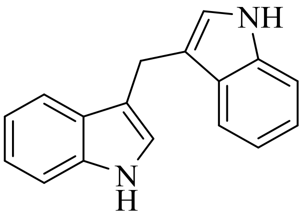 | -7.09 | -7.79 |
| **1-tosyl-I3C** | 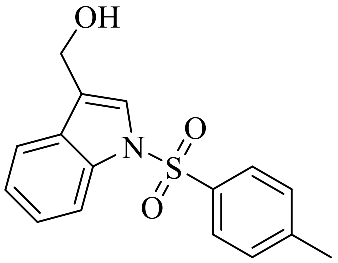 | -8.44 | -7.28 |

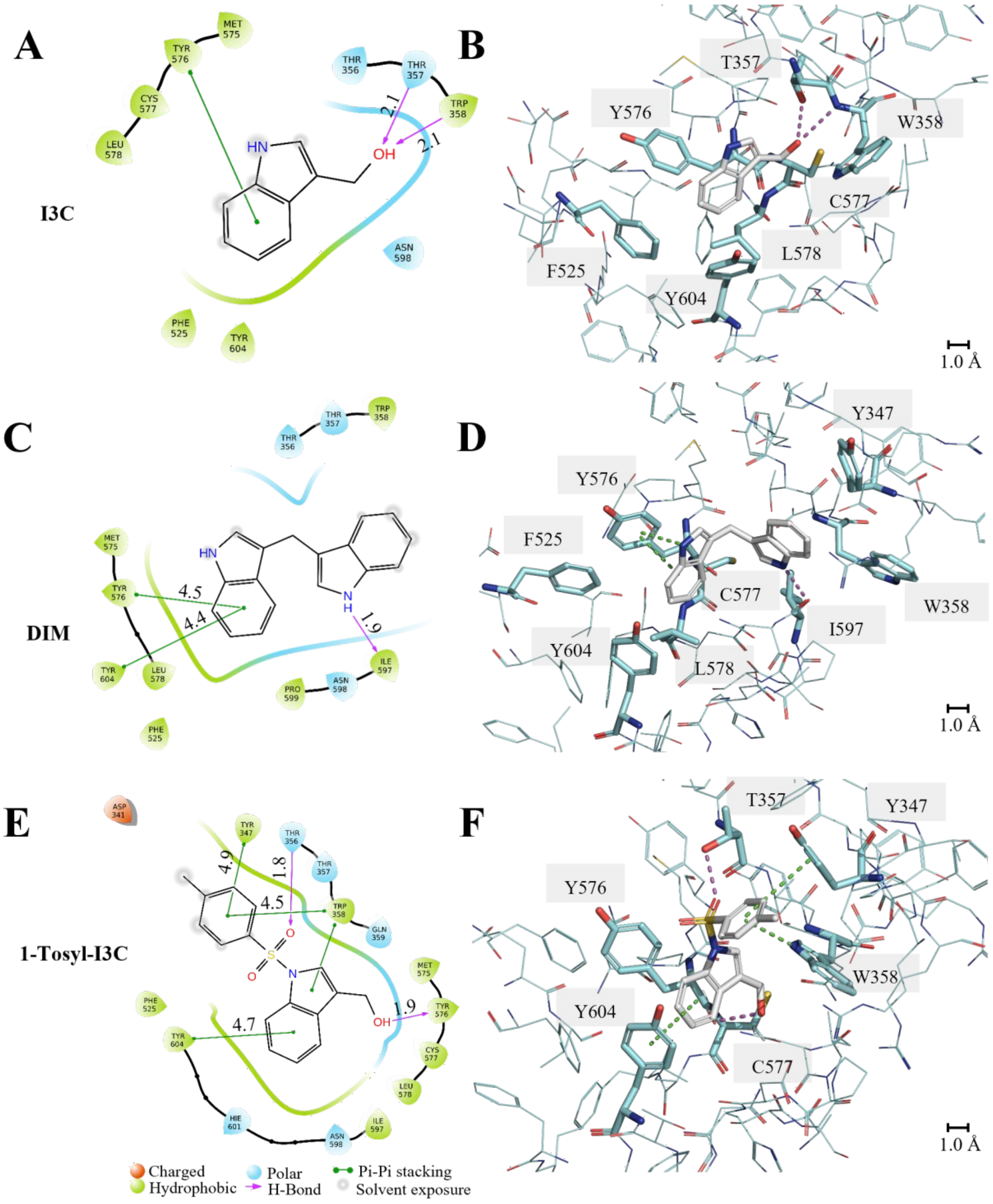


**Figure S5.** Ligand poses of selected compounds with WWP2. (A, B) I3C, (C, D) DIM and (E, F) 1-tosyl-I3C. Compounds were minimised and re-docked into the exo Ub site of PDB entry 6J1Z using Glide software. Hydrophobic (green), hydrophilic (blue) and charged (orange) residues are shown, with key polar (purple) and π-π (green) interactions given in Angstroms (Å). Panels A, C and E show the 2D interaction interfaces taken from Maestro. Panels B, D and F show 3D interaction interfaces with hydrogens removed. Images were created in PyMOL with key residues represented as sticks.

## Additional Biological Experimental Section

### *Materials*

All reagents were purchased from ThermoFisher, Sigma Aldrich and Melford, unless otherwise stated. All plasmids were either purchased from Addgene or kindly gifted (Table S3).

### *DNA Techniques*

Plasmids (Table S3) were transformed using standard heat shock or electroporation, incubating for 12-18 hours at 37 °C on LB agar plates containing respective antibiotics (Table S4).

**Table S3.** Plasmids, constructs, and their origins.

| Plasmid | Construct | Origin |
|---|---|---|
| **pET3a**-hUba1$^{His}$ | Wildtype | Addgene plasmid #63571[1] |
| **pGEX4T-1**-hUba1$^{GST\text{-}F2}$ | Wildtype | Gifted by Dr Arthur L Haas's Lab |
| **pET3a**-UbcH7$^{His}$ | Wildtype | Gifted by Professor Martin Scheffner |
| **pGEX4T-1**-UbcH7a$^{GST\text{-}F2}$ | Wildtype | Gifted by Dr Arthur L Haas's Lab |
| **pET32a**-WWP1-2L34H$^{His\text{-}Trx\text{-}3C}$ | WW2-2,3-linker-WW3-WW4-HECT | Gifted by Dr Wenyu Wen |
| **pET32a**-WWP1-L34H$^{His\text{-}Trx\text{-}3C}$ | 2,3-linker-WW3-WW4-HECT | Gifted by Dr Wenyu Wen |
| **pET28a**-WWP1-H$^{His\text{-}F2}$ | HECT | Dr Andrew Chantry's Lab |
| **pGEX2T**-WWP2-FL$^{GST\text{-}F2}$ | Wildtype | Dr Andrew Chantry's Lab |
| **pGEX6p-2**-WWP2-LH$^{GST\text{-}3C}$ | WW2-2,3-linker-HECT | Gifted by Professor Philip Cole |
| **pET28a**-WWP2-H$^{His\text{-}F2}$ | HECT | Dr Andrew Chantry's Lab |

### *Protein Purification Techniques*

All protocols were performed on ice or at 5 °C unless stated otherwise.

*Protein Expression*

Transformed E. coli cells were inoculated and incubated overnight at 37 °C, 180 rpm in LB containing appropriate antibiotics. The desired recombinant proteins were expressed, inducing with IPTG at $OD^{600}$ 0.6 – 1.0 before incubating at protein specific conditions (Table S4). Cells were pelleted by centrifugation (Beckman Coulter J20, JLA 8.1000 rotor) at 4,000 g, 4 °C for 30 minutes and stored at -20 °C.

**Table S4.** *E. coli* cell-line and protein expression conditions.

| Recombinant Protein | *E. coli* cell-line | [IPTG] (mM) | Temp (°C) | Antibiotics[(a)] |
|---|---|---|---|---|
| **hUba1**$^{His}$ | BL21-Star (DE3) | 1.0 | 25 | Amp |
| **hUba1**$^{GST\text{-}F2}$ | BL21 (DE3) | 1.0 | 25 | Amp |
| **UbcH7**$^{His}$ | BL21-CodonPlus RP | 1.0 | 25 | Amp, Cam |
| **UbcH7a**$^{GST\text{-}F2}$ | BL21 (DE3) | 0.4 | 20 | Amp |
| **WWP1-2L34H**$^{His\text{-}Trx\text{-}3C}$ | BL21-CodonPlus RP | 0.5 | 16 | Amp, Cam |
| **WWP1-L34H**$^{His\text{-}Trx\text{-}3C}$ | BL21-CodonPlus RP | 0.5 | 16 | Amp, Cam |
| **WWP1-H** $^{His\text{-}F2}$ | BL21-CodonPlus RP | 1.0 | 30 | Amp, Cam |
| **WWP2-FL**$^{GST\text{-}3C}$ | BL21-CodonPlus RP | 0.75 | 30 | Amp, Cam |
| **WWP2-LH**$^{GST\text{-}3C}$ | BL21-CodonPlus RP | 0.5 | 16 | Amp, Cam |
| **WWP2-H** $^{His\text{-}F2}$ | BL21-Rosetta1 | 1.0 | 25 | Kan, Cam |

(a) 50 ug/mL Amp (Ampicillin or Carbenicillin) and Kan (Kanamycin), 34 ug/mL Cam (Chloramphenicol).

***Protein Purification***

Protein specific buffers for each purification steps are shown in Table S5.

General protocols: Cells were lysed by either using a 4710 series ultrasonic homogenizer CP50 (Cole-Parmer) at 50 % amp for 10 seconds on, 10 seconds off for a total of 6 minutes or french pressed at 16,000 psi using a pre-cooled pressure cell (Thermo French Press). Affinity columns (Cytiva Life Sciences) were installed onto a

bench-top peristaltic pump (Parnachia Biotech) at 20 °C or AKTA pure 2 system (Cytiva Life Sciences) at 5 °C with supplier protocols followed. Sample concentrating was achieved using 5 or 10 kDa MW cut off Vivaspin protein concentrators (GE Healthcare), centrifuged (Beckman Coulter J-15R, JS-4.750 rotor) at 4,000 g for 10 to 20 minutes per spin with mixing. All samples were snap-frozen and stored at -80 °C unless otherwise stated.

His-tagged Uba1 and UbcH7: Pelleted cells from 1 L culture were resuspended in 35 mL buffer before being lysed, clarified, and purified through a 5 mL HisTrap™ FF column via straight elution. These were dialysed at 10 kDa cut-off in a 5 L reservoir overnight before concentrating and stored.

GST-tagged Uba1: Pelleted cells from 1 L culture were resuspended in 35 mL buffer before being lysed, clarified, and purified through a 1 mL GSTrap™ HP column via straight elution. The sample was then dialysed at 3 kDa cut-off in a 2 L reservoir overnight before concentrating and stored.

GST-tagged UbcH7: Pelleted cells from 2 L culture were resuspended in 35 mL buffer before being lysed, clarified, and loaded onto a 1 mL GSTrap™ HP column. On-column cleavage was achieved by incubating with 20 units per mL of thrombin overnight. Untagged UbcH7 was collected by washing the column with 7 CV of high salt PBS (500 mM NaCl) before passing through a 1 mL HiTrap Benzamidine column (Cytiva). The sample was then concentrated and stored.

His-tagged WWP1-2L34H: Pelleted cells from 8 L culture were resuspended in 70 mL buffer before being lysed, clarified, and purified through a 5 mL HisTrap™ FF column, washing for 20 CV before finally gradient eluting over 10 CV. The sample was then

dialysed at 10 kDa cut-off in a 5 L reservoir overnight and filtered to remove any precipitation. PreScission protease was added at 1.5 units per mg and incubated during another round of dialysis. The protease was then removed by reverse IMAC, before the sample was concentrated to 2 mL and loaded onto SEC 200 pg. Eluted fractions were pooled and concentrated for either immediate use in crystallography or stored.

His-tagged WWP1-L34H: Pelleted cells from 8 L culture were resuspended in 70 mL buffer before being lysed, clarified, and purified through a 5 mL HisTrap™ FF column, washing for 20 CV before finally eluting stepwise at 2 CV steps. The sample was then dialysed at 10 kDa cut-off in a 5 L reservoir overnight, filtering any precipitation before spin concentrating (10 °C) to 2 mL for gel filtration on a SEC 75 pg. PreScission protease was added at 2 units per mg to the eluted fractions and incubated overnight before removing by reverse GSTrap™. Buffer exchange was performed using the HiPrep™ 26/10 Desalting column to remove EDTA, followed by a reverse IMAC. The sample was concentrated to 2 mL and a second gel filtration was carried out on the SEC 75 pg. Eluted fractions were pooled and concentrated and stored.

His-tagged WWP1-H and WWP2-H: Pelleted cells from 1 L culture were resuspended in 35 mL buffer before being lysed, clarified, and purified through a 5 mL HisTrap™ FF column, washing for 10 CV before finally eluting via a gradient. To chelate leaked nickel, 1 mM EDTA was added before dialysis. The sample was then dialysed at 10 kDa cut-off in a 5 L reservoir overnight before spin concentrating for snap freezing and storage.

GST-tagged WWP2-LH: Pelleted cells from 4 L culture were resuspended in 35 mL buffer before being lysed, clarified, and purified through a 5 mL GSTrap™ FF column

on bench top, collecting the straight elution on ice. PreScission protease was added at 1 unit per mg, and incubated during dialysed at 10 kDa cut-off in a 5 L reservoir overnight, before being refreshed for a further 4 hrs. A reverse GSTrap was used, removing protease, uncleaved WWP2-LH and free GST. The sample was concentrated to 2 mL for gel filtration on a SEC 75 pg. Eluted fractions were pooled and again passed through the reverse GSTrap before adding 5 % glycerol. Samples were concentrated at 20 °C for either immediate use in crystallography or stored.

**Table S5.** Proteins and their respective buffers.

| Recombinant Protein | Buffer | Contents |
|---|---|---|
| **hUba1**$^{His}$ | Resuspension & Wash | 20 mM $Na_2PO_4$ pH 7.4, 500 mM NaCl, 20 mM Imidazole containing 1 x Roche tablet |
| | Elution | 20 mM $Na_2PO_4$ pH 7.4, 500 mM NaCl, 500 mM Imidazole |
| | Dialysis | 50 mM Tris.HCl pH 7.4, 1 mM DTT |
| **hUba1**$^{GST-F2}$ | Resuspension & Wash | 10 mM $Na_2HPO_4$ pH 7.4, 2 mM $KH_2PO_4$, 2.7 mM KCl, 137 mM NaCl containing 1 x Roche tablet |
| | Elution | 10 mM $Na_2HPO_4$ pH 7.4, 2 mM $KH_2PO_4$, 2.7 mM KCl, 137 mM NaCl, 50 mM Reduced Glutathione |
| | Dialysis | 10 mM $Na_2HPO_4$ pH 7.4, 2 mM $KH_2PO_4$, 2.7 mM KCl, 137 mM NaCl |
| **UbcH7**$^{His}$ | Resuspension & Wash | 20 mM $Na_2PO_4$ pH 7.4, 500 mM NaCl, 20 mM Imidazole containing 1 x Roche tablet |
| | Elution | 20 mM $Na_2PO_4$ pH 7.4, 500 mM NaCl, 500 mM Imidazole |
| | Dialysis | 50 mM HEPES pH 7.4, 150 mM NaCl, 1 mM DTT |
| **UbcH7a**$^{GST-F2}$ | Resuspension & Wash | 10 mM $Na_2HPO_4$ pH 7.4, 2 mM $KH_2PO_4$, 2.7 mM KCl, 137 mM NaCl containing 1 x Roche tablet |
| | Elution | 10 mM $Na_2HPO_4$ pH 7.4, 2 mM $KH_2PO_4$, 2.7 mM KCl, 500 mM NaCl |
| **WWP1-2L34H**$^{His-Trx-3C}$ | Resuspension & Wash | 100 mM Tris pH 8.0, 250 mM NaCl, 10 mM imidazole containing 0.1 mM PMSF |
| | Elution | 100 mM Tris pH 8.0, 250 mM NaCl, 250 mM imidazole |
| | Dialysis | 100 mM Tris pH 8.0, 100 mM NaCl, 1 mM DTT. 1 mM EDTA, 25 mM Maltose |
| | SEC | 50 mM Tris pH 8.0, 500 mM NaCl, 1 mM DTT, 1 mM EDTA |
| **WWP1-L34H**$^{His-Trx-3C}$ | Resuspension | 50 mM Tris pH 8.0, 500 mM NaCl, 10 mM imidazole containing 0.1 mM PMSF |
| | Wash | 50 mM Tris pH 8.0, 500 mM NaCl, 30 mM imidazole |
| | Elution | 50 mM Tris pH 8.0, 500 mM NaCl, 250 mM imidazole |
| | Dialysis & SEC | 50 mM Tris pH 8.0, 100 mM NaCl, 1 mM DTT, 1 mM EDTA |
| | Buffer Exchange | 50 mM Tris pH 8.0, 500 mM NaCl, 1 mM DTT |
| | Second SEC | 50 mM Tris pH 8.0, 500 mM NaCl, 1 mM DTT, 1 mM EDTA |
| **WWP1-H_**$^{His-F2}$ & **WWP2-H_**$^{His-F2}$ | Resuspension | 50 mM Tris.HCl pH 8.0, 500 mM NaCl, 2 mM DTT, 10 mM Imidazole, 5 % Glycerol containing 0.1 mM PMSF |
| | Wash | 50 mM Tris.HCl pH 8.0, 500 mM NaCl, 2 mM DTT, 25 mM Imidazole, 5 % Glycerol |
| | Elution | 50 mM Tris.HCl pH 8.0, 500 mM NaCl, 2 mM DTT, 300 mM Imidazole, 10 % Glycerol |
| | Dialysis | 50 mM Tris.HCl pH 8.9, 150 mM NaCl, 5 mM DTT |
| **WWP2-LH**$^{GST-3C}$ | Resuspension | 25 mM Tris.HCl pH 8.0, 250 mM NaCl containing 0.1 mM PMSF and 1 x Roche tablet. |
| | Wash | 25 mM Tris.HCl pH 8.0, 250 mM NaCl, 0.1 % Triton X-100 |
| | Elution | 25 mM Tris.HCl pH 8.0, 250 mM NaCl, 50 mM reduced glutathione. |
| | Dialysis | 25 mM Tris.HCl pH 8.0, 250 mM NaCl, 5 mM DTT |
| | SEC | 25 mM Tris.HCl pH 7.5, 150 mM NaCl, 5 mM DTT or 3 mM TCEP |

***SDS-PAGE Analysis***

Samples were collected at relevant purification stages and diluted to the appropriate concentration for SDS-PAGE analysis using Bolt$^{Tm}$ pre-cast 4 – 12 % BIS-Tris Plus acrylamide gels. Manufactures protocols followed combining sample, 1 × Bolt$^{Tm}$ LDS Buffer and 1 × Bolt$^{Tm}$ Reducing Agent before heating in a thermal cycler at 75 °C for 10 minutes and running gels in the Invitrogen Mini Gel Tank using 1 × Bolt$^{Tm}$ MES SDS Buffer at 165 V for 30 minutes. InstantBlue (Expedeon) was used to stain gels overnight before de-staining and storing in $H_2O$.

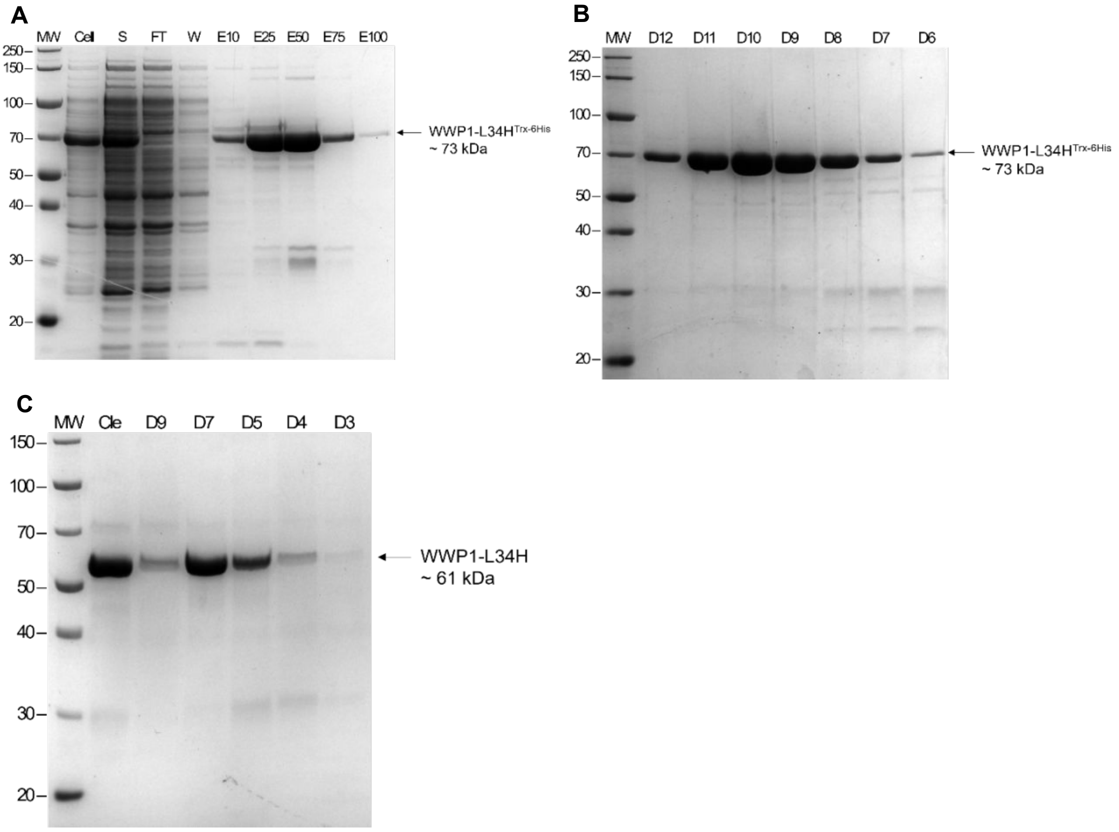


**Figure S6.** SDS-PAGE analysis of WWP1-L34H purification. (A) Stepwise IMAC isolation. Intense band at ~73 kDa indicating Trx-His tagged WWP1-L34H. Lane labels: Ladder (MW), whole cell (Cell), soluble lysate (S), column flow-through (FT), column wash (W), 10% elution (E10), 25% elution (E25), 50% elution (E50), 75% elution (E75), 100% elution (E100). All fractions were pooled. (B) First SEC purification. Trx-His tagged WWP1-L34H identified as shown by band at ~73 kDa. Lanes labelled as column fraction positions excluding ladder (MW). (C) Cleavage and second SEC purification. Intense band at ~61 kDa as untagged WWP1-L34H. Lanes labelled as fraction positions other than ladder (MW) and cleavage sample (Cle). Fractions D9 – D4 were pooled. Image enhanced using Image Lab (BioRad).

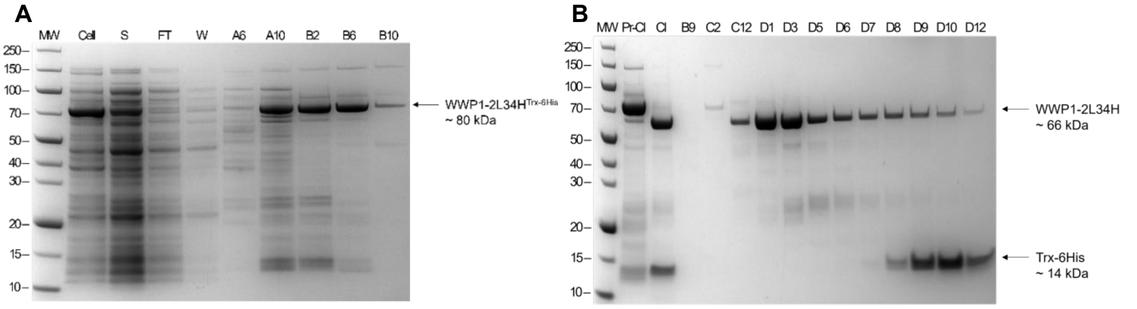


**Figure S7.** SDS-PAGE analysis of WWP1-2L34H purification. (A) Gradient IMAC isolation. Intense bands at ~80 kDa indicating Trx-His tagged WWP1-2L34H. Lane labels: Ladder (MW), whole cell (Cell), soluble lysate (S), column flow-through (FT), column wash (W), elution fractions positions (A6 – B10). Fractions A10 – B10 were pooled. (B) SEC purification. Untagged WWP1-2L34H identified by intense bands at ~66 kDa. Lanes labelled as fraction positions other than ladder (MW), pre-cleavage (Pr-Cl) and cleavage sample (Cl). Fractions C12 – D6 were pooled. Image enhanced using Image Lab (BioRad).

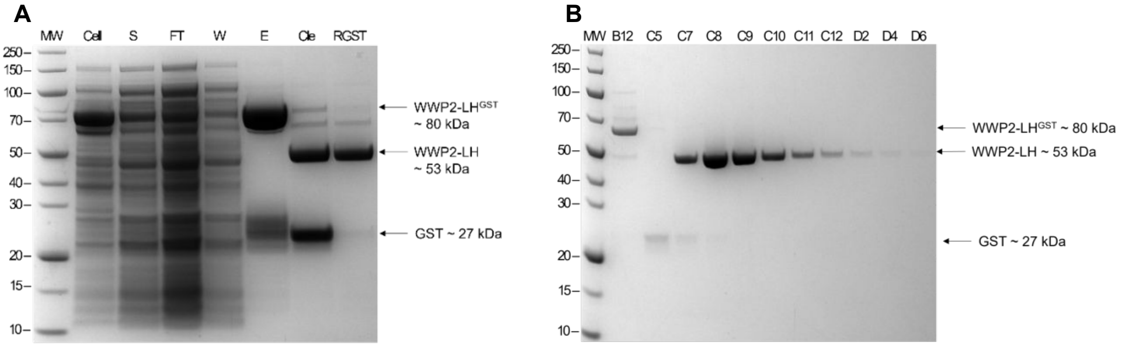


**Figure S8.** SDS-PAGE analysis of WWP2-LH purification. (A) Straight GSTrap isolation and cleavage. Isolation and cleavage of GST-tagged WWP2-LH shown by intense band at ~80 kDa and ~53 kDa respectively, with free GST shown at ~27 kDa band. Lane labels: Ladder (MW), whole cell (Cell), soluble lysate (S), column flow-through (FT), column wash (W), column elution (E), cleavage sample (Cle) and reverse GSTrap flow-through (RGST). (B) SEC purification. Untagged WWP2-LH identified by intense bands at ~53 kDa. Lanes labelled as fraction positions, with fractions C7 – D6 pooled. Image enhanced using Image Lab (BioRad).

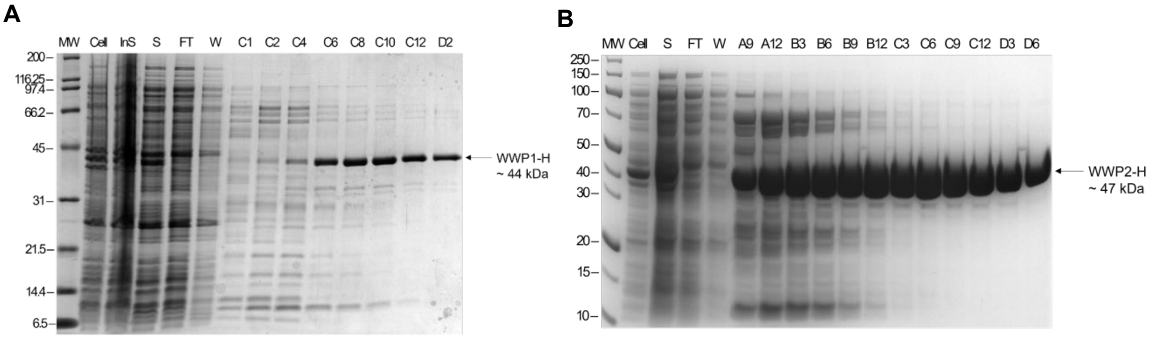


**Figure S9.** SDS-PAGE analysis of WWP1-HECT and WWP2-HECT purification. (A) Gradient IMAC isolation of WWP1-HECT. Intense bands at ~44 kDa indicating His tagged WWP1-H. Lane labels: Ladder (MW), whole cell (Cell), insoluble (InS), soluble lysate (S), column flow-through (FT), column wash (W), elution fractions positions (C1 – D2). Fractions C6 – D2 were pooled. (B) Gradient IMAC isolation of WWP2-HECT. Intense bands at ~44 kDa indicating His tagged WWP2-H. Lane labels: Ladder (MW), whole cell (Cell), soluble lysate (S), column flow-through (FT), column wash (W), elution fractions positions (A9 – D6). Fractions C6 – D6 were pooled. Image enhanced using Image Lab (BioRad).

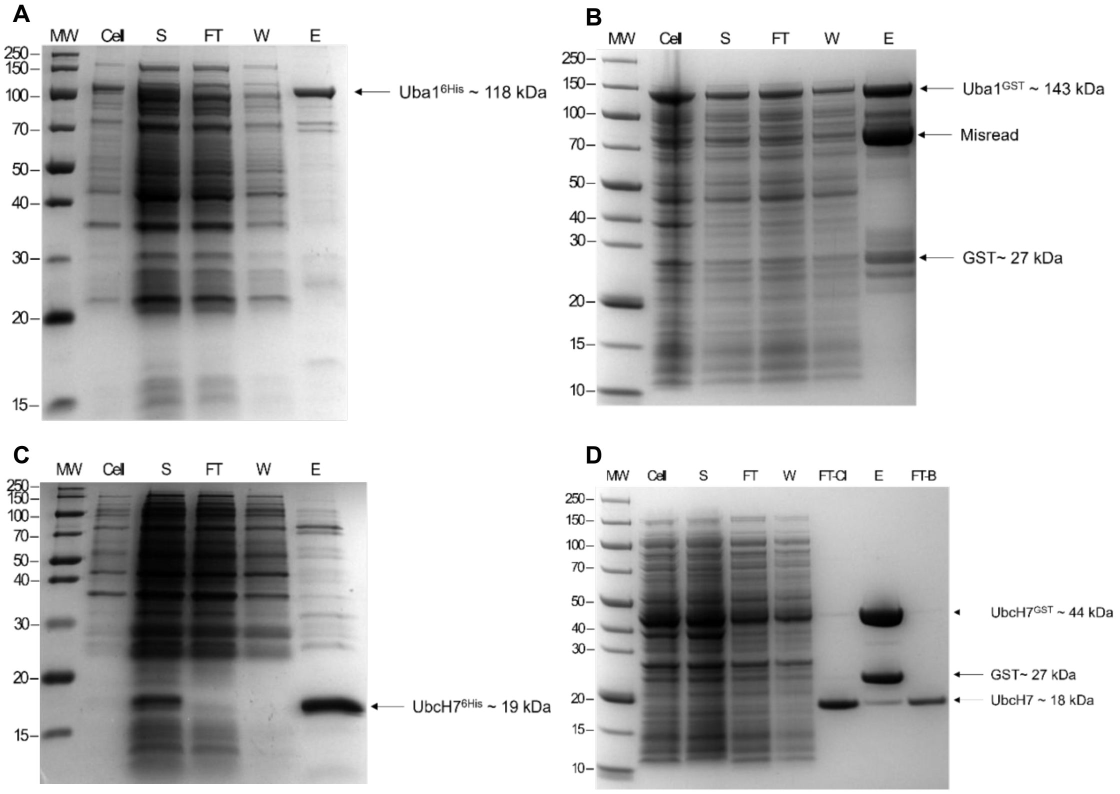


**Figure S10.** SDS-PAGE analysis of Uba1 and UbcH7 purifications. Lane labels: Ladder (MW), whole cell (Cell), soluble lysate (S), column flow-through (FT), column wash (W), column elution (E), on-column cleavage flow-through (FT-Cl) and Benzamidine column flow-through (FT-B). (A) Straight IMAC isolation of Uba1. Intense band at ~118 kDa for His-tagged Uba1. (B) Straight GST affinity isolation of Uba1. Intense bands at ~ 143 kDa for GST-tagged Uba1, as well as possible misread at ~80 kDa. (C) Straight IMAC isolation of UbcH7. Intense band at ~19 kDa for His-tagged UbcH7. (D) Straight GST affinity isolation of UbcH7 and cleavage. Intense bands at ~44 kDa for GST-tagged UbcH7, ~ 27 kDa for free GST and 18 kDa for untagged UbcH7. Image enhanced using Image Lab (BioRad).

***Differential Scanning Fluorimetry (DSF)***

A 96 well-plate (MicroAmp Optical) was loaded with 18 μL of 3.8 μM WWP1-2L34H and 2.5 μM WWP2-LH, in their respective buffers (SI Table S5) containing 5 × SYPRO orange dye. A 2 μL aliquot of the compound was added to a final concentration of 100 μM containing 0.1 % DMSO before the plate was sealed (MicroAmp Clear Adhesive Film). Both non-protein and DMSO controls were also generated. The plates were briefly centrifuged before the assay was run using an ABI 7500 RT-PCR following the melt curve using ROX™ (575 nm) as the 'pre-set' fluorescence dye. A standard thermal profile of 25 – 70 °C, rising at 0.5 °C per minute was used. The mid-point melting temperature (Tm) was calculated using a Boltzmann fit to the fluorescence curve using Protein Thermal Shift Software v1.4 (ThermoFisher). Results were further processed and plotted using Excel.

***Auto-ubiquitination Assay***

Cell lysate containing His-tagged WWP1-L34H or GST-tagged WWP2-FL proteins were incubated on either 96-well Clear Pierce glutathione [2] or nickel-coated plates for 1 hour. Reaction mixtures of either 3 ng/well GST-Uba1 and 15 ng/well UbcH7 or 10 ng/well His-Uba1 and 150 ng/well His-UbcH7 were incubated together in 25 mM Tris pH 8.0, 100 mM NaCl, 4 mM $MgCl_2$ containing 60 ng/well FLAG-ubiquitin and 1.25 mM ATP for 40 minutes. A prior 1 % BSA plate blocking step is required for nickel-coated plates. After plate washing, 2 μL of the compound was added at the desired concentration (1 % DMSO) followed by 18 μL of the reaction mixture. This was then incubated for 2 hours with 0% and 100% controls before 100 μL of anti-FLAG M2-Peroxidase HRP (1: 10,000 PBST) was added to each well and incubated for 1 hour. Finally, 100 μL of 1 × TMB substrate solution (Invitrogen) was added to each well and

incubated for up to 10 minutes until sufficient blue colour change was observed. To stop the reaction, 100 μL of 1 M HCl was added. For the counter assay, 3 ng/well GST-Uba1 and 200 ng/well UbcH7 were incubated with the other reaction mixture components for 1 hour before incubating onto plates for a further 1 hour. All other steps were followed. The plates were washed three times with PBST (and 15 mM Imidazole) between each step. Quantification was measured by absorbance read at 450 nm. All assay optimisations have been previously reported by Watt and colleagues[3].

Relative activity (RA) was determined by comparing the autoubiquitination of the WWP ligases in the presence of each compound, i.e TMB absorbance (450 nm), to the 100 % controls (1 % DMSO only), further normalising against background absorbance using 0 % controls (no E3 loaded).

$$Relative\ Acitivty\ (\%) = \frac{X\ (\mathrm{A}) - 0\%(\mathrm{A})}{100\%(\mathrm{A}) - 0\%\ (\mathrm{A})} \times 100$$

Where $X$ = Compound; 100% = 1% DMSO control; 0% = No E3 control.

$IC_{50}$ non-linear regression curves were calculated in GraphPad v10.2 (Prism).

***Saturation Transfer Difference (STD) NMR***

An Amicon centrifuge filter unit with a 10 kDa MW cutoff was used to exchange the protein in 25 mM $d_{19}$-2,2-bis(hydroxymethyl)-2,2′,2″-nitrilotriethanol, 100 mM NaCl and 1.0 mM DTT buffer pH* 8.9 (uncorrected for the deuterium isotope effect on the pH glass electrode) in $D_2O$. The STD NMR sample was composed of 500 μM indol-3-carbinol and 20 μM protein (WWP1 and WWP2, respectively). For all STD NMR

experiments, the on- and off-resonance spectra were acquired using a train of 50 ms Gaussian selective saturation pulses using a variable saturation time, with on-resonance frequency at 0.0 ppm and off-resonance frequency at 40 ppm. The binding epitope mapping determination (STD build-up curves) was obtained at incremental saturation times from 0.5 to 5 s. Residual protein resonances were filtered out using a $T_2$ filter of 40 ms. All the STD NMR experiments were performed with a spectral width of 10 kHz and 32768 data points using 256 or 512 scans. All the NMR experiments were performed on a Bruker Avance 800.23 MHz at 278 K. Binding epitope mappings were obtained by determining the initial slopes ($STD_0$) calculated by performing a least-squares fitting of the following mono-exponential curve:

$$STD(t_{sat}) = STD_{max}(1 - \exp(k_{sat} * t_{sat}))$$

where $STD(t_{sat})$ is the STD intensity for a saturation time, $t_{sat}$, $STD_{max}$ is the maximum STD intensity and $k_{sat}$ is the rate constant for saturation transfer. In the limit, $t_{sat}$→:

$$STD_0 = STD_{max} * k_{sat}$$

Importantly, $STD_0$ gives a value that is independent of any relaxation or rebinding effects, allowing for a more accurate binding epitope. The value of $STD_0$ was then normalized against the proton with the largest intensity to give values in the range of 0–100%, which were then mapped onto the ligand structure to give the corresponding binding epitope mapping.

***Molecular Docking***

Molecular docking was performed using the Schrödinger Suite 2020-3. The protein structures of NEDD4 HECT (PDB ID: 5C91)[4], as well as WWP1 (PDB ID: 9EQK) and WWP2 (PDB ID: 6J1Z)[5] both containing the HECT and the WW2 domains, were prepared using the Schrödinger's Protein Preparation Wizard module (Epik v5.5, Impact v8.8)[6,7]. I3C, DIM, as well as 1-tosyl-I3C and compound 16, were prepared using LigPrep v5.5 (Epik v5.3)[7]. Default settings were used for both proteins and ligands at pH 7.0 ± 0.2, removing all waters and adding hydrogen atoms. Both WWP1 and WWP2 structures and ligands were aligned to NEDD4 and its covalent I3C analogue, before performing minimisation to both the ligand and residues surrounding an 8 Å radius. This was achieved using the OPLSe force field in MacroModel v12.9 at a default 2500 iterations[8–14]. The ligands were then re-docked into the minimised pseudo-bound structures using the Glide SP v8.8 program with grids generated from the individual minimised ligand positions[15,16]. Default settings were used with the top five poses generated, enabling post-ligand minimisation before being ranked and binding affinity given as GlideScore. Figures were created in 2D using the Schrodinger Ligand Interaction Diagram module, with 3D molecular models generated using PyMOL v2.5[17]

**Additional Chemical Experimental Section**

Unless specified, all reagents and starting materials were purchased from commercial sources (Sigma-Aldrich (Merck Life Sciences), Fluorochem (Doug Discovery), Fischer Scientific, Alfa Aesar) and used as supplied. Indole-3-carbinol was purchased from Fluorochem and used as received (97% purity). Thin-layer chromatography was performed on Merck silica gel 60 F254 plates and visualised by UV absorption, purchased from VWR International. Flash column chromatography was carried out using Silica Gel 60 purchased from Material Harvest. 'Concentrated' refers to the removal of volatile organic solvents *via* distillation using a rotary evaporator. 'Dried' refers to pouring onto or adding anhydrous $MgSO_4$ or $Na_2SO_4$ to (as specified), followed by filtration. Water refers to deionised water.

NMR spectra were recorded on 400 or 500 MHz Bruker NMR spectrometer using the deuterated solvent stated in the reported data. $^{1}H$, $^{13}C$ and $^{19}F$ NMR samples were prepared by dissolving a sample in 0.4 mL – 0.7 mL deuterated solvent. All deuterated solvents were purchased from Cambridge Isotopes and used as received, solvents were stored under 4 Å molecular sieves after opening. All spectra were referenced to the residual solvent peaks of the solvent used.[2] NMR spectra chemical shifts ($\delta$) are reported in ppm and coupling constants ($J$) are reported in hertz (Hz). Abbreviations for NMR splitting are s (singlet), d (doublet), t (triplet), q (quartet), and m (multiplet). Infrared spectra were recorded using a Perkin Elmer Spectrum Two LITA. High-resolution mass spectrometry was performed at the University of East Anglia using a UPLC-HRMS (ACQUITY H-Class PLUS UPLC and Waters SYNAPT XS High Resolution Mass Spectrometer) setup with electrospray ionisation using ca. 1 $\mu gmL^{-1}$ solution in acetonitrile or methanol. Melting points (not corrected) were

recorded on a Büchi Melting Point B-545 using capillary melting point tubes made in-house. Compounds **7**, **9**, **10**, **11**, **12**, **13**, **14**, **15**, **16** and **DIM** were synthesised and are all 95 – 99% purity as determined by 1H NMR analysis. Oxime **7** existed as an approx. 1:1 mixture of *cis* and *trans* isomers.

***General procedure 1:*** *PTC sulfonamide formation*

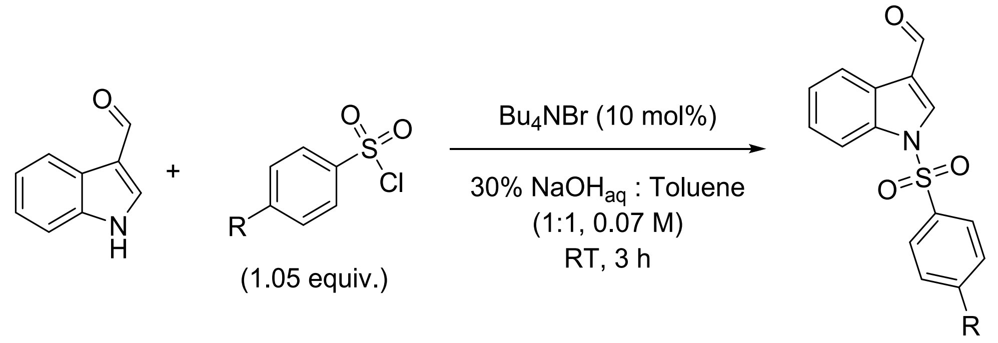


To an RBF was added indole-3-carboxaldehyde and toluene (0.14 M). To the stirred suspension was added 30% NaOH solution (0.14 M) and $Bu_4NBr$ (10 mol%). Afterwards, the respective sulfonyl chloride (1.05 equiv.) was added, and the mixture was vigorously stirred for 3 h. The mixture was transferred to a sep. funnel and separated, the aqueous phase was washed with toluene (20 mL). The organic layers were collected and dried ($MgSO_4$) and solvent removed. Purified by column chromatography to provide the respective *N*-(arylsuflonyl)jndole-3-carboxaldehyde. Adapted from literature conditions[18].

***General procedure 2:*** *N-(aryl)indole-3-carboxaldehyde reduction*

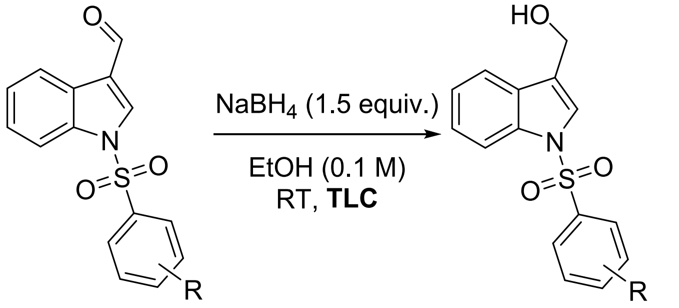


To a vial was added the respective *N*-functionalised indole-3-carboxaldehyde and ethanol (0.1 M). To the stirred solution was added $NaBH_4$ (1.5 equiv.) and allowed to stir at RT, monitored by TLC. $H_2O$ (3 mL) was added to quench the reaction after completion, the mixture was transferred to a separatory funnel and brine was added (10 mL), the mixture was extracted with EtOAc (3x 20 mL), the organic layers collected and washed with brine (10 mL), dried ($MgSO_4$) and the solvent removed under reduced pressure at 27 - 28 $^{o}$C. Purified by column chromatography (7:3 Hexane : EtOAc), followed by trituration with hexane.

***Compound Synthesis***

***Compound 13A*** – *N-benzylindole-3-carboxaldehyde*

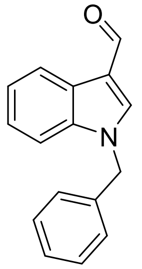

To a 50 mL RBF was added indole-3-carboxaldehyde (200 mg, 1.38 mmol), MeCN (13.8 mL, 0.1 M) and $CsCO_3$ (372.6 mg, 1.0 equiv.). Heated to reflux for 2 h. Afterwards, benzyl bromide (0.18 mL, 1.32 equiv.) was added *via* syringe and the mixture continued to reflux for 1 h. After completion of the reaction by TLC the mixture was allowed to cool to RT and the solvent was removed under reduced pressure. The residue was taken up in water (50 mL) and extracted with EtOAc (3x 50 mL). The organic layers were collected and dried ($MgSO_4$), solvent was removed *in vacuo.* The residue was purified by column chromatography (8:2 Hexane : EtOAc) to provide *N*-benzylindole-3-carboxyaldehyde as a white solid (312 mg, 1.33 mmol, 96%).

$^1$H NMR (400 MHz, C*D*$Cl_3$) $\delta$ 10.01 (s, 1H), 8.36 – 8.31 (m, 1H), 7.72 (s, 1H), 7.37 – 7.29 (m, 6H), 7.21 – 7.17 (m, 2H), 5.37 (s, 2H).

$^{13}$C NMR (101 MHz, C*D*$Cl_3$) $\delta$ 184.6, 138.4, 137.5, 135.3, 129.2, 128.4, 127.2, 125.5, 124.2, 123.1, 122.2, 118.6, 110.4, 51.0.

IR (cm$^{-1}$): 2815 (C-H aldehyde), 1650 (C=O).

Followed literature procedure, data matches literature[19].

NMR Data:

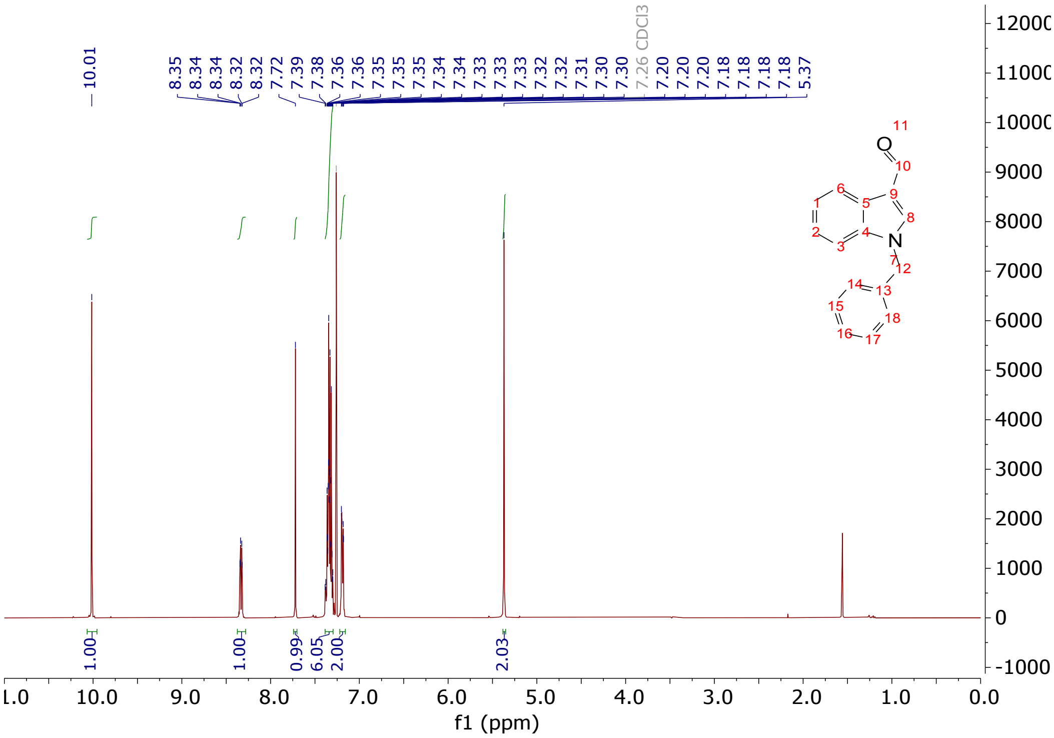


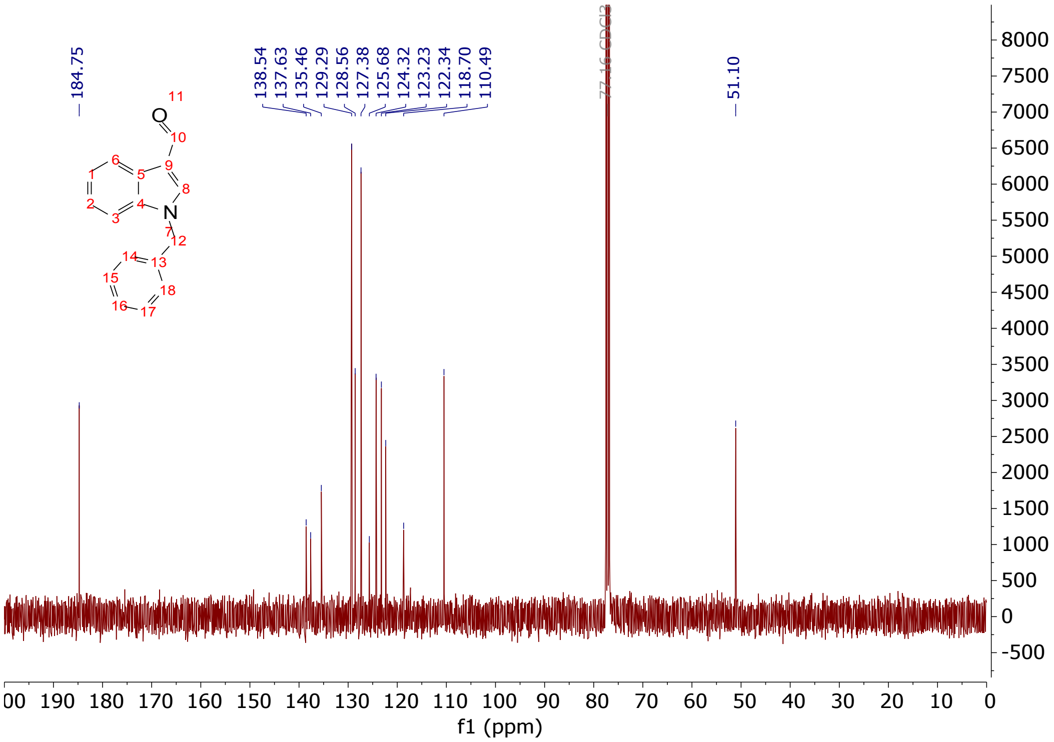


**Figure S11.** NMR data for **Compound 13A** – N-benzylindole-3-carboxaldehyde

***Compound 15A*** – *N-(4-methylbenzenesulfonyl)indole-3-carboxaldehyde*

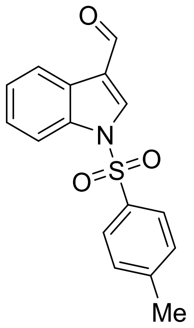

Followed general procedure 1. Indole-3-carboxaldehyde (200 mg, 1.37 mmol), toluene (9.84 mL), 30% NaOH soln. (9.84 mL), $Bu_4NBr$ (44.4 mg), tosyl chloride (275.8 mg). Purified by column chromatography (9:1 Hex. : EtOAc) to provide *N*-(4-methylbenzenesulfonyl)indole-3-carboxaldehdye as a white solid (293 mg, 0.98 mmol, 75%).

$^1$H NMR (400 MHz, C*D*$Cl_3$) $\delta$ 10.10 (s, 1H), 8.25 (ddd, *J* = 7.5, 1.6, 0.8 Hz, 1H), 8.23 (s, 1H), 7.97 – 7.93 (m, 1H), 7.88 – 7.83 (m, 2H), 7.44 – 7.39 (m, 1H), 7.36 (td, *J* = 7.6, 1.2 Hz, 1H), 7.32 – 7.27 (m, 2H), 2.38 (s, 3H).

$^{13}$C NMR (101 MHz, C*D*$Cl_3$) $\delta$ 185.5, 146.3, 136.3, 135.4, 134.5, 130.5, 127.4, 126.5, 125.2, 122.8, 122.5, 113.4, 21.8 (13 out of 14 carbon resonances found).

IR (cm$^{-1}$): 3133, 2847 (C-H aldehyde), 1661 (C=O).

Data in line with literature data[20].

NMR Data:

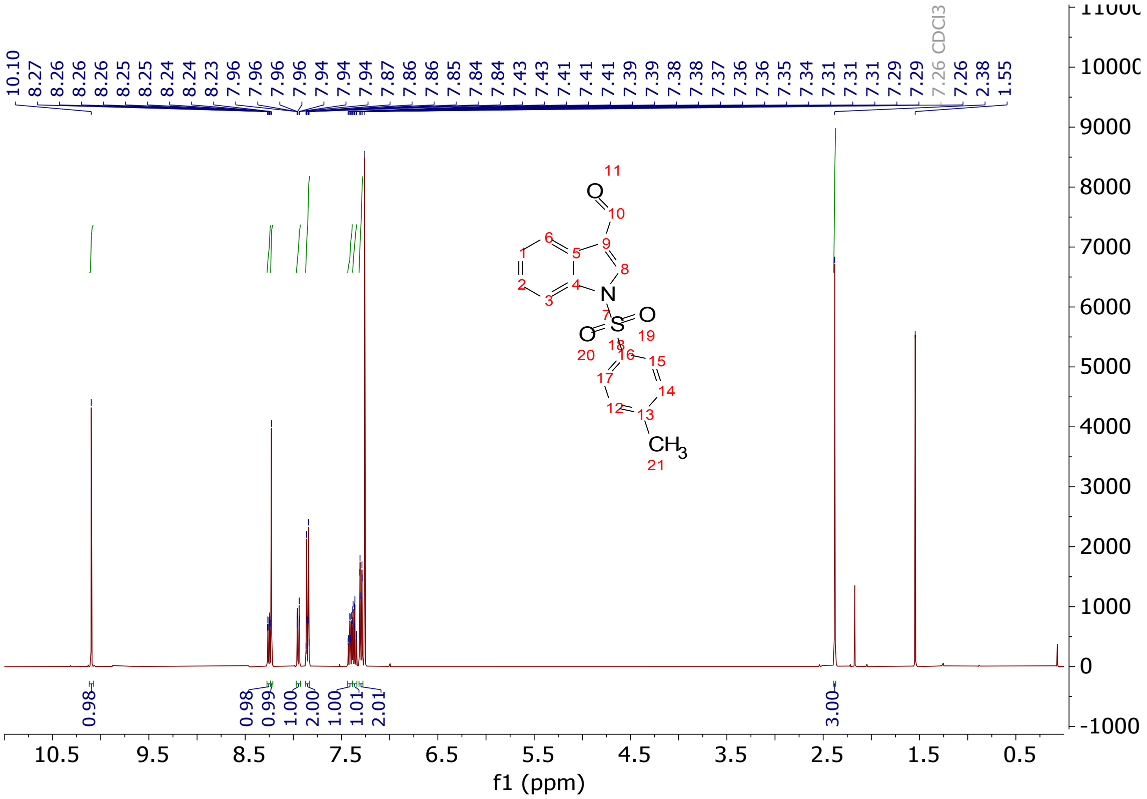

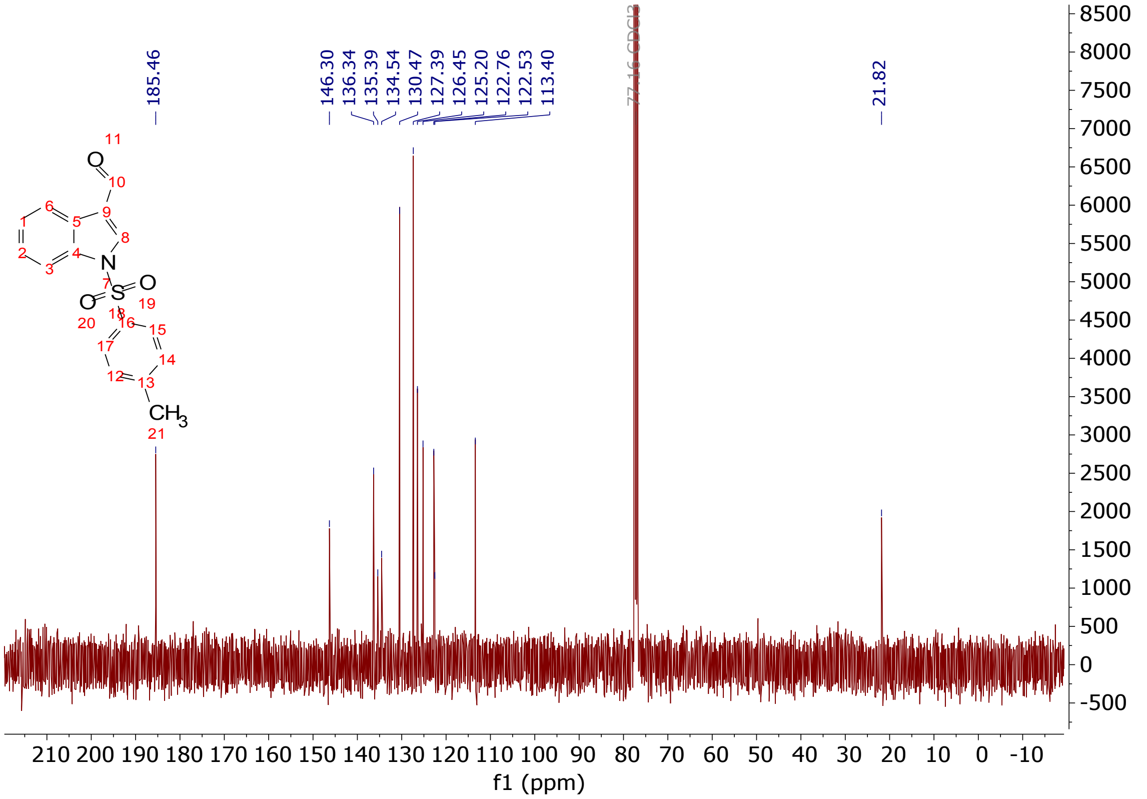

**Figure S12.** NMR data for **Compound 15A** – N-(4-methylbenzenesulfonyl)indole-3-carboxaldehyde

***Compound 16A*** *– N-(4-trifluoromethylbenzenesuflonyl)indole-3-carboxaldehyde*

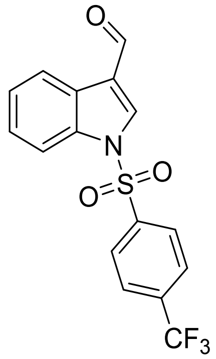


Prepared by following general procedure 1. Indole-3-carboxaldehyde (282.1 mg, 1.94 mmol), toluene (6.9 mL), 30% NaOH solution (9.2 mL), $Bu_4NBr$ (62.5 mg) 4-trifluoromethylbenzenesulfonyl chloride (500 mg, 2.04 mmol). Column chromatography (9:1 Hex. : EtOAc) provided *N*-(4-trifluoromethylbenzenesuflonyl)jndole-3-carboxaldehyde as a white solid (0.260 g, 0.74 mmol, 38%).

$^{1}$H NMR (400 MHz, Acetone-*d*$_6$) $\delta$ 10.16 (s, 1H), 8.73 (s, 1H), 8.39 (d, *J* = 8.3 Hz, 2H), 8.22 (dt, *J* = 7.8, 1.0 Hz, 1H), 8.06 (dt, *J* = 8.3, 1.0 Hz, 1H), 8.02 (d, *J* = 8.1 Hz, 2H), 7.49 (ddd, *J* = 8.4, 7.3, 1.4 Hz, 1H), 7.42 (td, *J* = 7.5, 1.1 Hz, 1H).

$^{13}$C NMR (101 MHz, Acetone-*d*$_6$) $\delta$ 186.7, 141.8, 138.3, 136.2, 136.0, 129.3, 128.1 (q, *J* = 3.8 Hz), 127.4 (d, *J* = 3.0 Hz), 126.2, 123.8, 123.3, 114.2 (12 out of 13 carbon resonances found).

$^{19}$F NMR (376 MHz, Acetone-*d*$_6$) $\delta$ -64.00.

IR (cm$^{-1}$): 3107, 2923 (C-H aldehyde), 1666 (C=O).

M.P. 124.9 – 126.1 $^{o}$C.

MS ES+ m/z Calc. for $C_{16}H_{10}F_3NO_3S$ $(M+H)^+$: 356.0403, Found: 356.0392.

NMR Data:

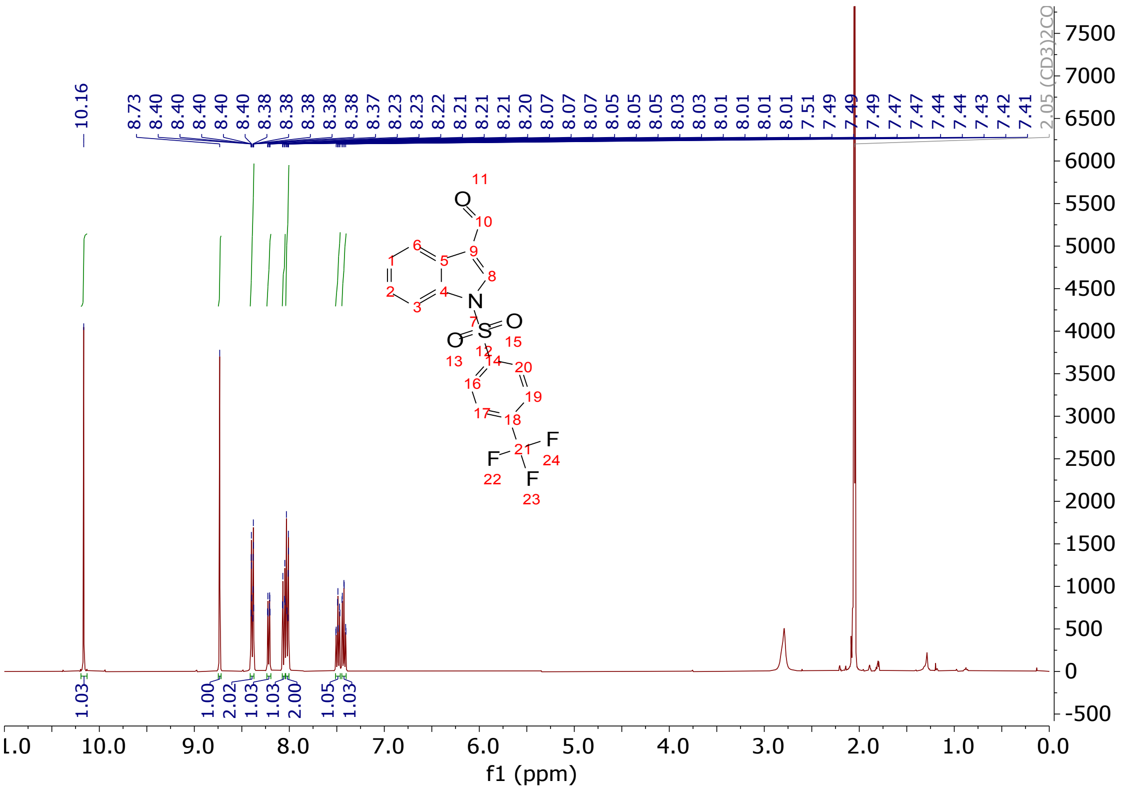

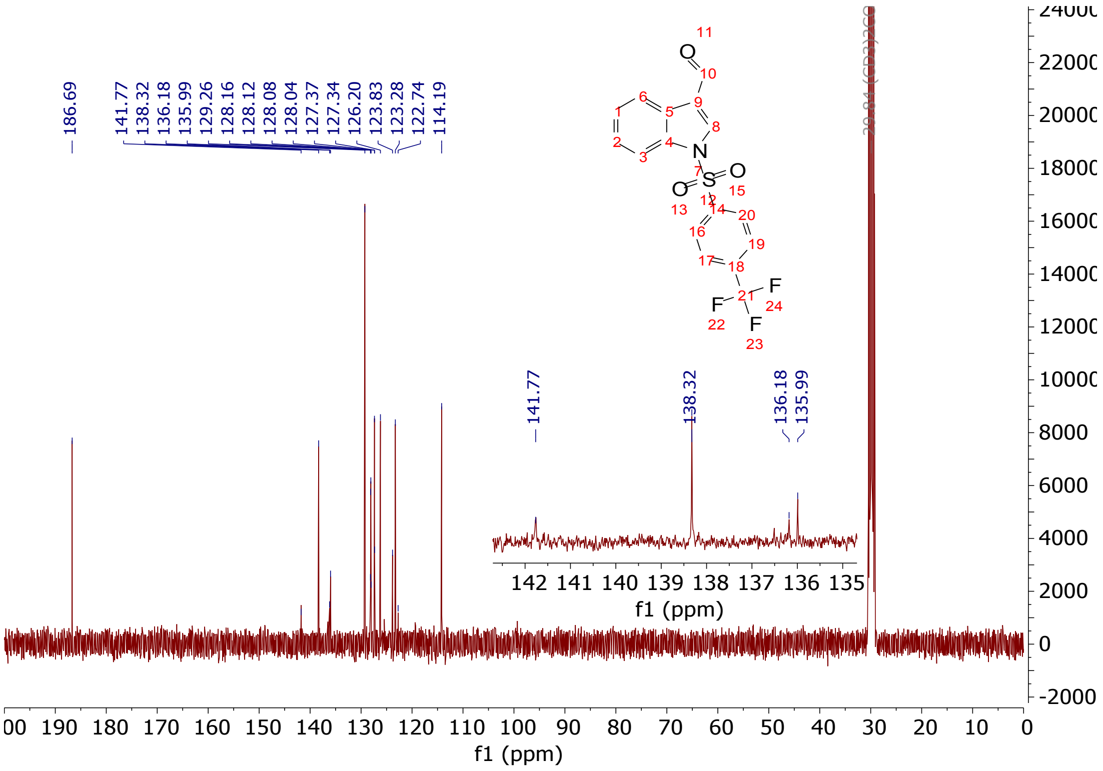

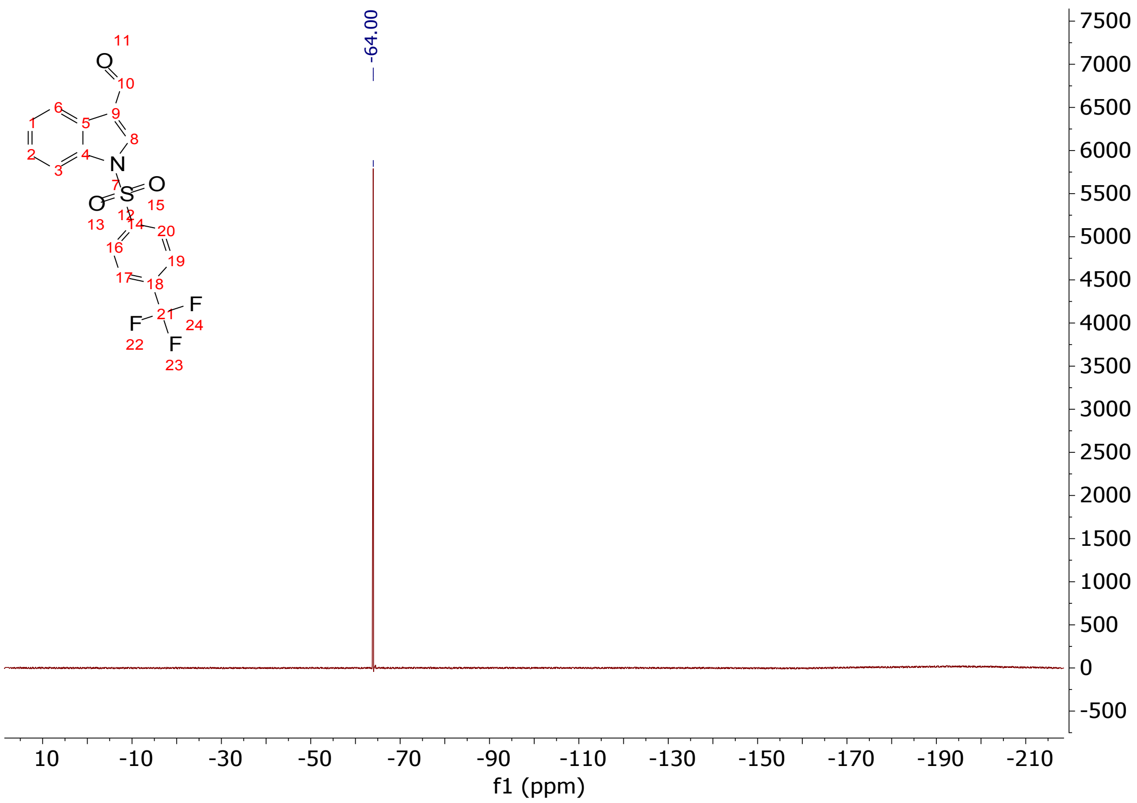

**Figure S13.** NMR data for **Compound 16A** – N-(4-trifluoromethylbenzenesuflonyl)indole-3-carboxaldehyde

HRMS Data:

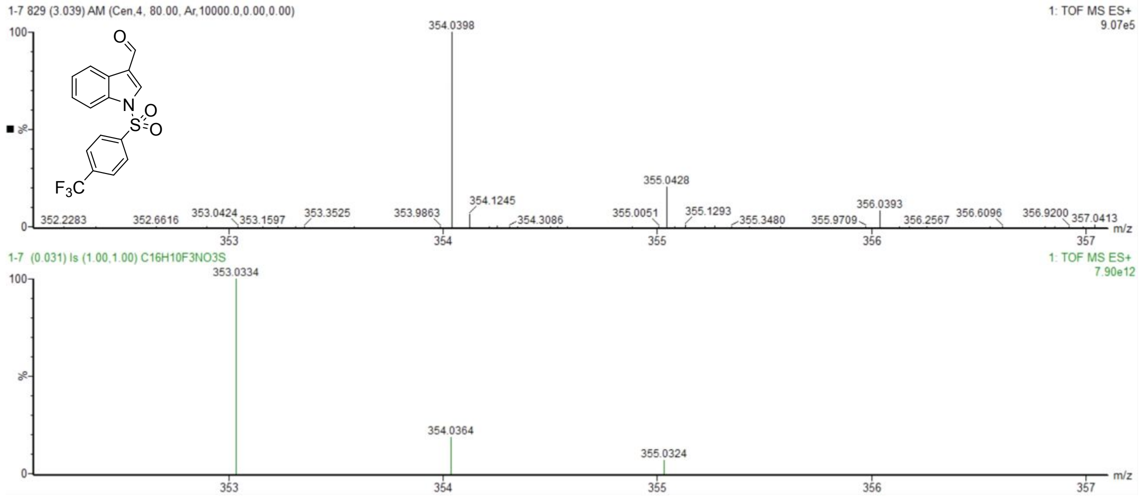

**Figure S14.** HRMS data for **Compound 16A** – N-(4-trifluoromethylbenzenesuflonyl)indole-3-carboxaldehyde

***Compound 13*** – *N-benzylindole-3-carbinol*

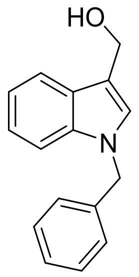


To a 25 mL RBF was added *N*-benzylindole-3-carboxaldehyde (300 mg, 1.275 mmol), distilled THF (6.4 mL) and EtOH (6.4 mL) to reach a total concentration of 0.1 M. $NaBH_4$ (62.7 mg, 1.3 equiv.) was added in one batch at 0 °C and the mixture allowed to warm to RT and stirred for 18 h. Afterwards, water (20 mL) was added to the suspension and the solvents were removed *in vacuo.* The aqueous suspension was then transferred to a separatory funnel and extracted with EtOAc (3x 20 mL). The organic layers were collected and washed with brine (20 mL), dried ($MgSO_4$) and solvent removed under reduced pressure to provide a white solid. Purified by column chromatography (8:2 -> 7:3 Hex. : EtOAc) to provide *N*-benzylindole-3-carbinol as a white solid (197 mg, 0.83 mmol, 65%).

$^1$H NMR (400 MHz, C*D*$Cl_3$) $\delta$ 7.75 (dt, *J* = 7.5, 1.0 Hz, 1H), 7.33 – 7.26 (m, 4H), 7.21 (ddd, *J* = 8.2, 7.0, 1.4 Hz, 1H), 7.19 – 7.15 (m, 1H), 7.15 – 7.11 (m, 3H), 5.30 (s, 2H), 4.89 (d, *J* = 5.5 Hz, 2H), 1.42 (d, *J* = 5.5 Hz, 1H).

$^{13}$C NMR (101 MHz, C*D*$Cl_3$) $\delta$ 137.4, 137.0, 129.0, 127.9, 127.4, 127.14, 127.08, 122.4, 119.9, 119.4, 115.6, 110.0, 57.4, 50.2.

IR (cm$^{-1}$): 3367 (OH).

Data matches that of the literature[21].

NMR Data:

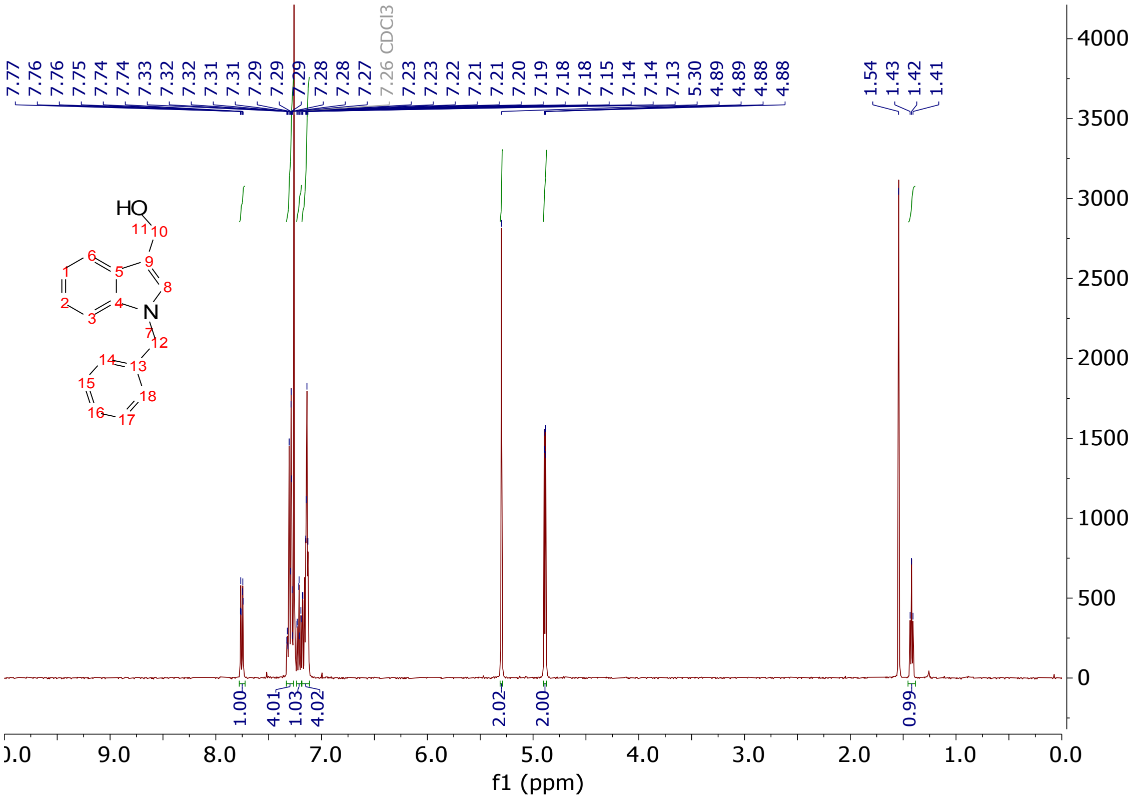


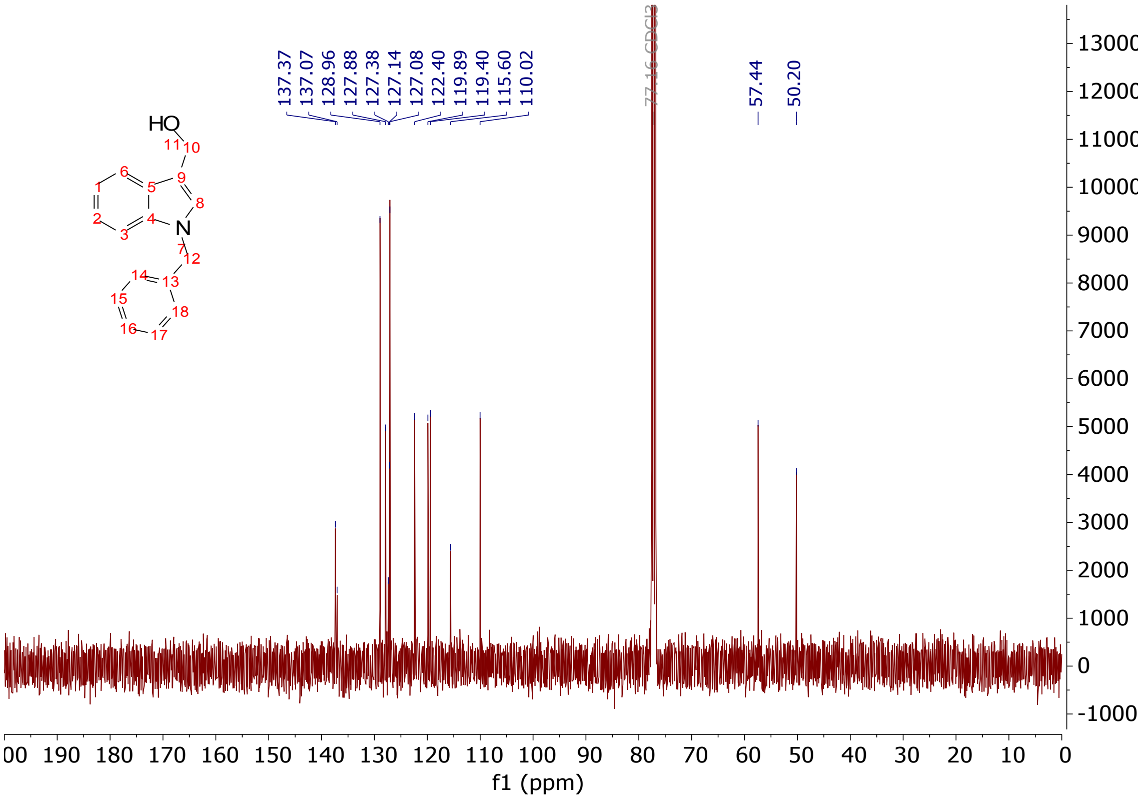


**Figure S15.** NMR data for ***Compound 13*** – *N-benzylindole-3-carbinol*

***Compound 15*** – *N-(4-methylbenzenesulfonyl)indole-3-carbinol*

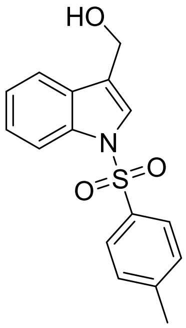


Adapted from general procedure 2. *N*-(4-methylbenzenesulfonyl)indole-3-carboxaldehyde (200 mg, 0.668 mmol), EtOH (6.68 mL, 0.1 M), sodium borohydride (38 mg, 1.5 equiv.). 5 h reaction time. Routine workup provided *N*-(4-methylbenzenesulfonyl)indole-3-carbinol as a colourless oil (168 mg, 0.56 mmol, 83%).

$^{1}$H NMR (400 MHz, C*D*Cl$_3$) $\delta$ 7.99 (dt, *J* = 8.4, 0.9 Hz, 1H), 7.81 – 7.73 (m, 2H), 7.61 (dt, *J* = 7.8, 1.1 Hz, 1H), 7.55 (d, *J* = 1.1 Hz, 1H), 7.34 (ddd, *J* = 8.4, 7.2, 1.3 Hz, 1H), 7.28 – 7.20 (m, 3H), 4.82 (dd, *J* = 5.7, 1.0 Hz, 2H), 2.34 (s, 3H), 1.59 (br t, *J* = 5.7 Hz, 1H).

$^{13}$C NMR (101 MHz, C*D*Cl$_3$) $\delta$ 145.2, 135.6, 135.4, 130.1, 129.6, 127.0, 125.2, 124.0, 123.5, 122.4, 120.0, 113.9, 57.4, 21.7.

IR (cm$^{-1}$): 3384 (OH).

Data in line with literature data[20].

NMR Data:

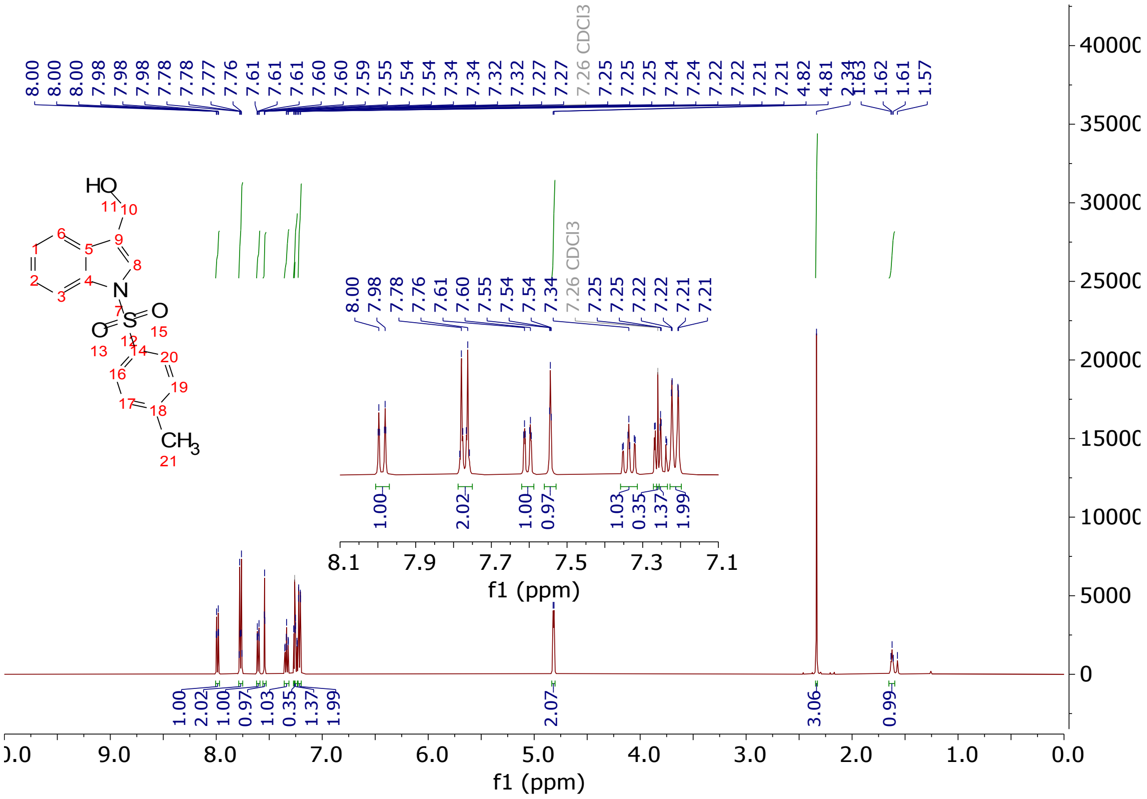

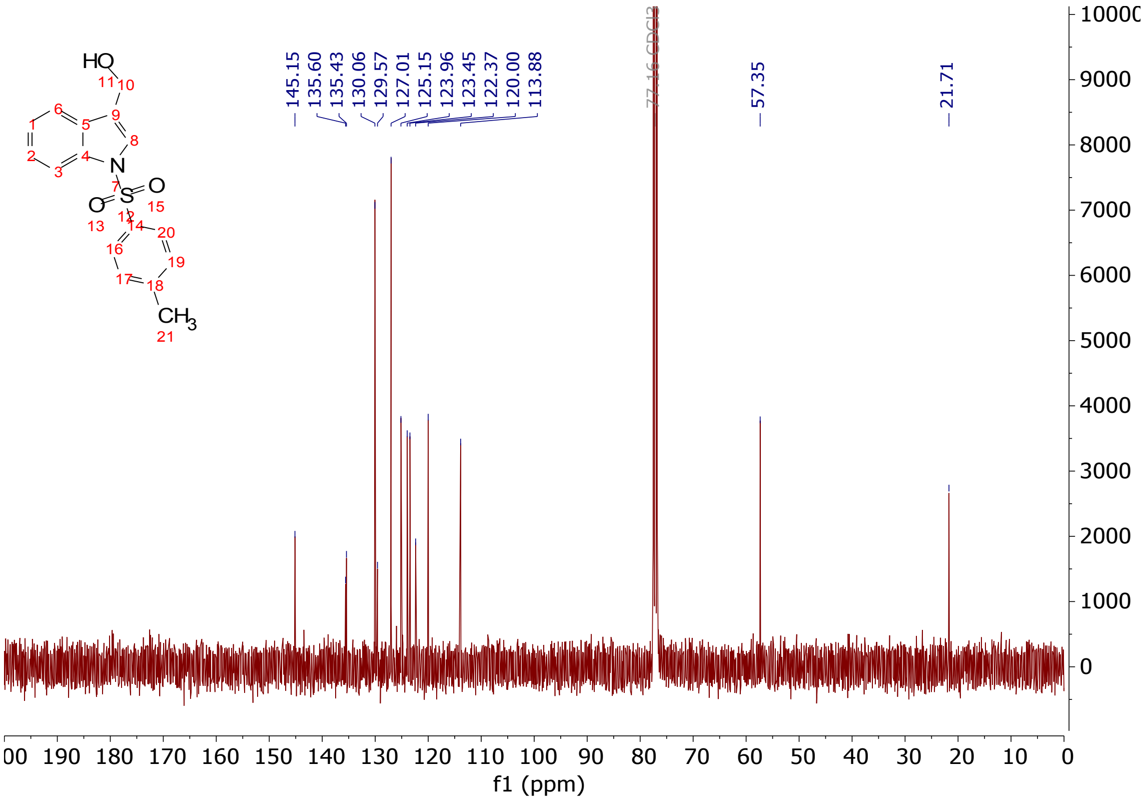

**Figure S16**. NMR data for **Compound 15** – N-(4-methylbenzenesulfonyl)indole-3-carbinol

***Compound 16*** – *N-(4-trifluoromethylbenzenesulfonyl)indole-3-carbinol*

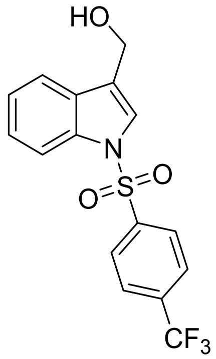

Followed general procedure 2. *N*-(4-trifluoromethylbenzenesulfonyl)indole-3-carboxaldehyde (30 mg, 0.085 mmol), $NaBH_4$ (5 mg, 1.5 equiv.), EtOH (1 mL). Reaction time 2 h. Provided an off-white solid of *N*-(4-trifluoromethylbenzenesulfonyl)indole-3-carbinol (30 mg, quant.).

$^1H$ NMR (400 MHz, C*D*$Cl_3$) $\delta$ 8.04 – 7.97 (m, 3H), 7.73 – 7.67 (m, 2H), 7.65 – 7.59 (m, 1H), 7.54 (d, *J* = 1.1 Hz, 1H), 7.38 (ddd, *J* = 8.5, 7.3, 1.3 Hz, 1H), 7.29 (ddd, *J* = 8.3, 7.3, 1.1 Hz, 1H), 4.84 (dd, *J* = 5.7, 1.1 Hz, 2H), 1.62 (t, *J* = 5.7 Hz, 1H).

$^{13}C$ NMR (101 MHz, C*D*$Cl_3$) $\delta$ 141.63 135.6, 135.4, 129.7, 127.5, 126.7 (q, J = 3.9 Hz), 125.6, 124.0, 123.6, 123.5, 120.3, 113.8, 57.3 (13 out of 14 carbon resonances observed).

$^{19}F$ NMR (376 MHz, C*D*$Cl_3$) $\delta$ -63.37.

IR ($cm^{-1}$): 3246 (OH).

M.P. 164.1 – 165.5 $^oC$.

MS ESI+ Calc. for $C_{16}H_{11}F_3NO_2S$ (-OH): 339.0493, found: 339.0493.

NMR Data:

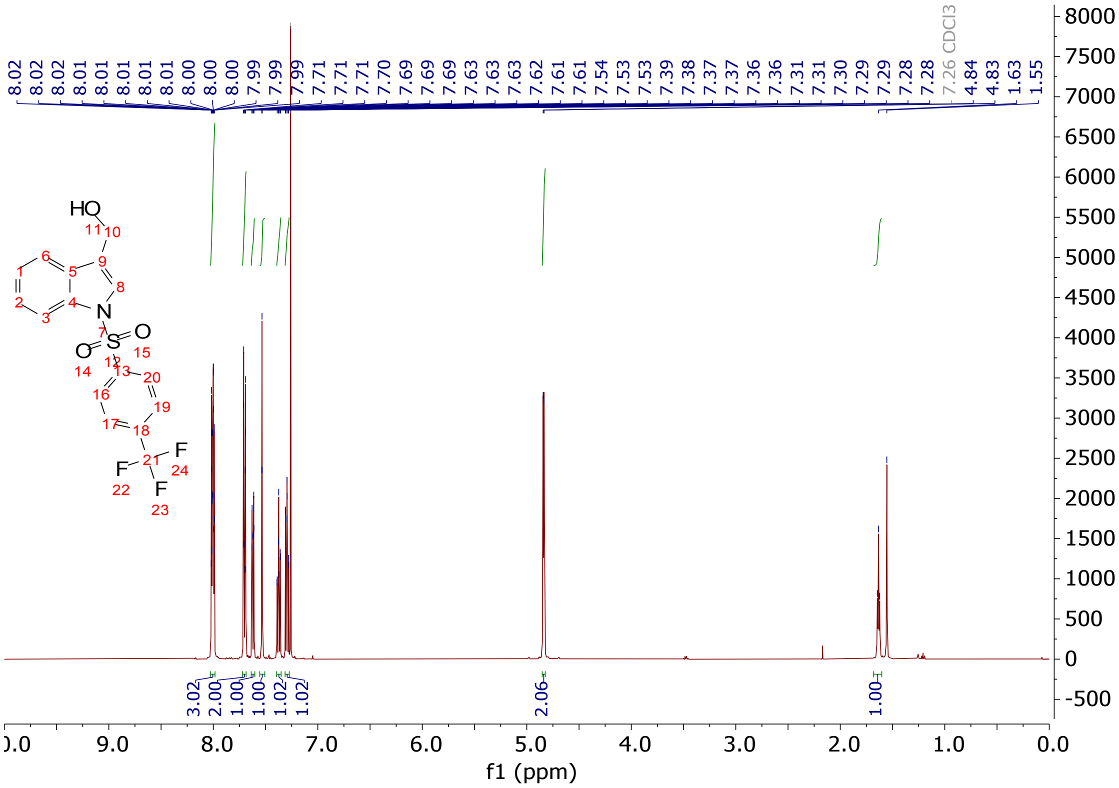
8.02 8.02 8.02 8.01 8.01 8.01 8.01 8.01 8.00 8.00 8.00 7.99 7.99 7.99 7.71 7.71 7.71 7.70 7.69 7.69 7.69 7.63 7.63 7.63 7.62 7.61 7.61 7.54 7.53 7.53 7.39 7.38 7.37 7.37 7.36 7.36 7.31 7.31 7.30 7.29 7.29 7.28 7.28 7.26 CDCl3 4.84 4.83 1.63 1.55
3.02 2.00 1.00 1.00 1.02 1.02 2.06 1.00
f1 (ppm)

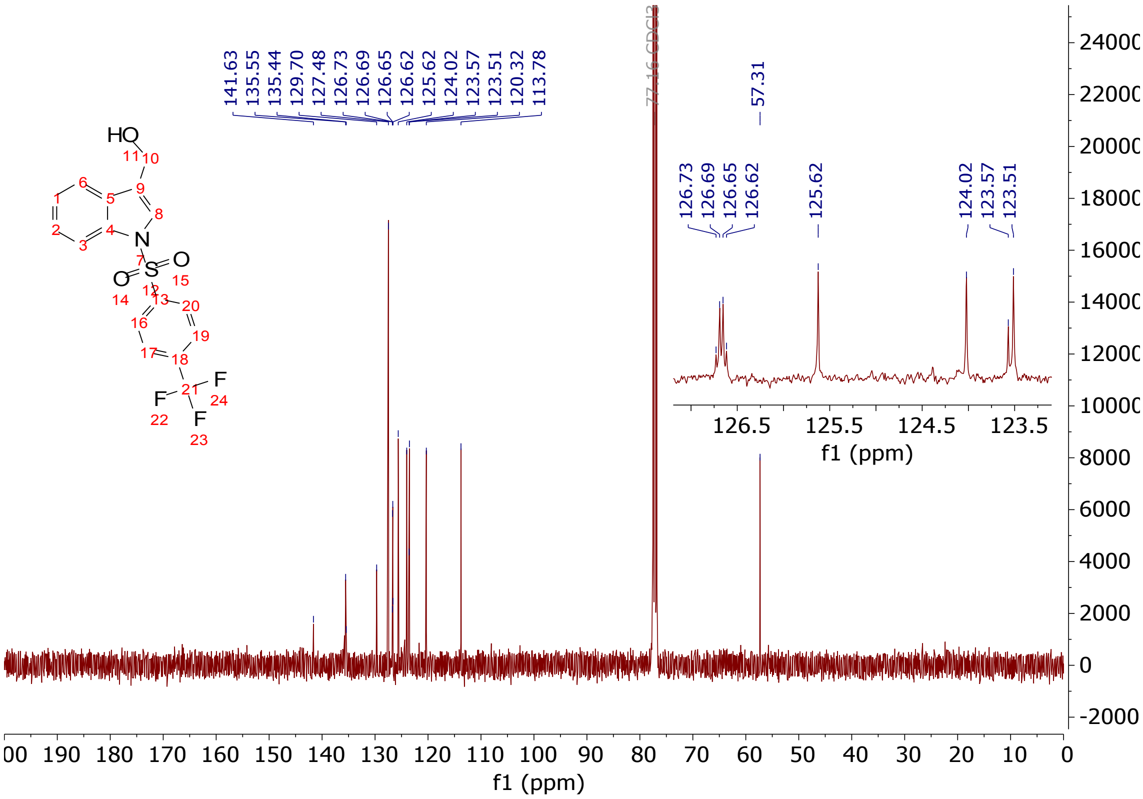
141.63 135.55 135.44 129.70 127.48 126.73 126.69 126.65 126.62 125.62 124.02 123.57 123.51 120.32 113.78
77.16 CDCl3
57.31
126.73 126.69 126.65 126.62 125.62 124.02 123.57 123.51
f1 (ppm)

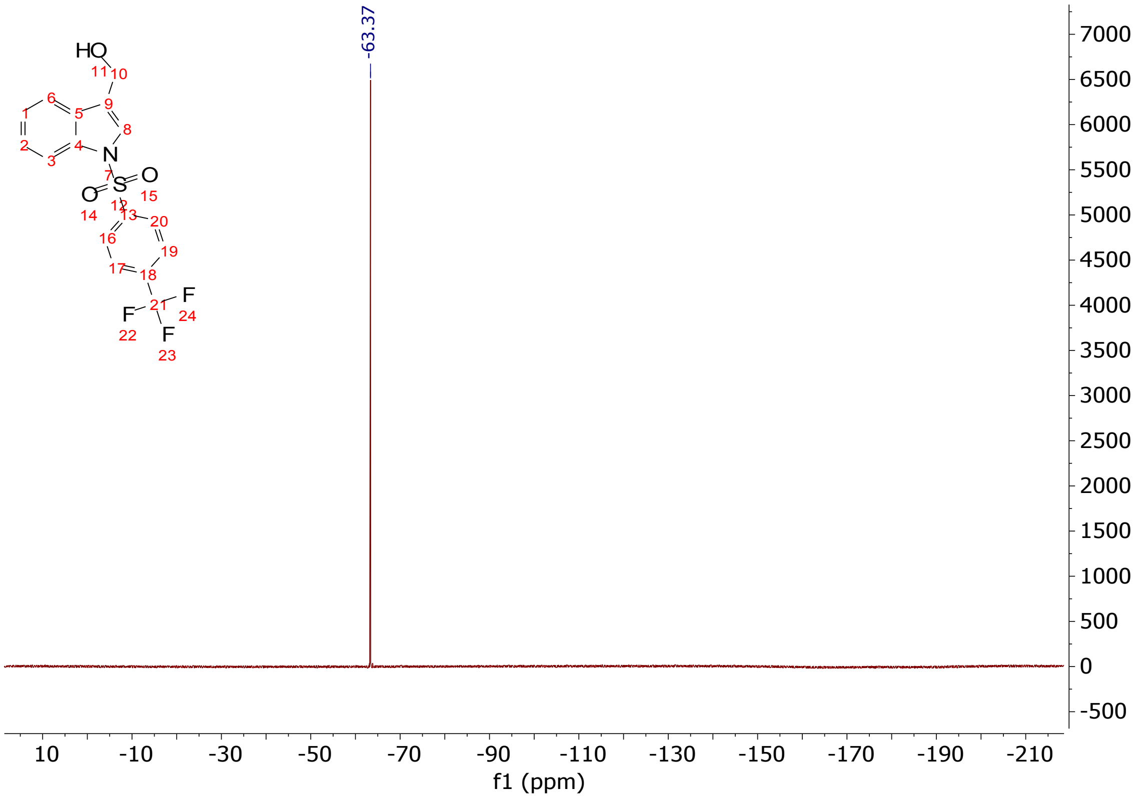


**Figure S17.** NMR data for **Compound 16** – N-(4-trifluoromethylbenzenesulfonyl)indole-3-carbinol.

HRMS Data:

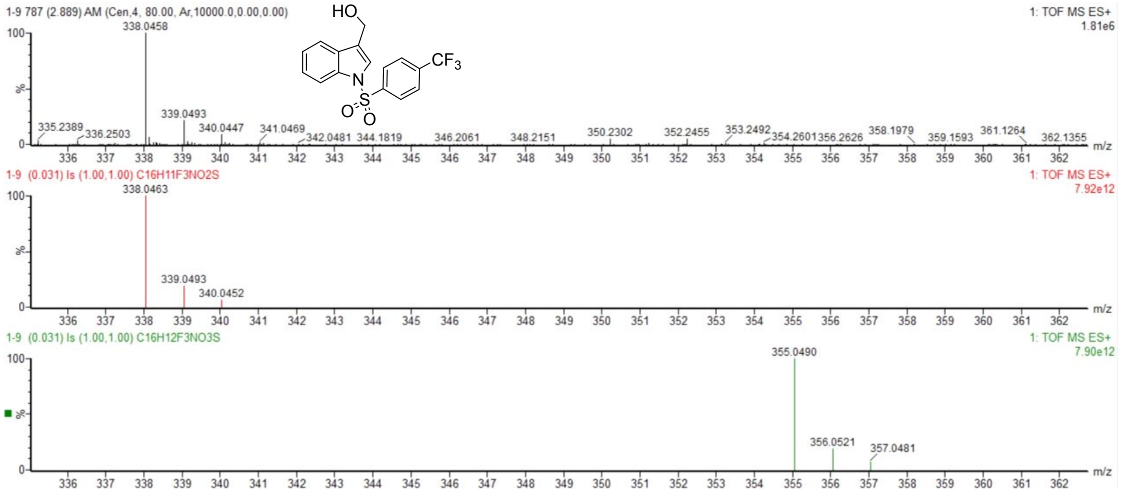


**Figure S18.** HRMS data for **Compound 16** – N-(4-trifluoromethylbenzenesulfonyl)indole-3-carbinol.

***DIM*** *– 3,3'-diindolylmethane*

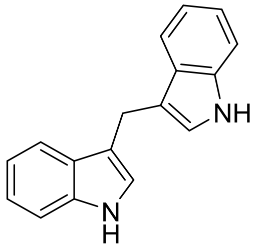

To a 250 mL RBF was added indole-3-carbinol (1.0 g, 6.78 mmol) and 2.0 M NaOH solution (100 mL, 0.068 M). The mixture was heated to reflux for 1 h. The mixture was cooled and neutralised using $CO_2$ gas (dry ice). The precipitate was filtered and dried, then subjected to column chromatography (8:2 Hex. : EtOAc) to provide 3,3'-diindolylmethane as an off-white solid (341 mg, 1.38 mmol, 20%).

$^1$H NMR (400 MHz, C*D*$Cl_3$) $\delta$ 7.90 (br s, 2H), 7.63 (dd, J = 8.0, 1.1 Hz, 2H), 7.36 (dd, J = 8.0, 1.1 Hz, 2H), 7.19 (ddd, J = 8.2, 7.0, 1.2 Hz, 2H), 7.10 (ddd, J = 8.0, 7.0, 1.0 Hz, 2H), 6.97 – 6.92 (m, 2H), 4.25 (s, 2H).

$^{13}$C NMR (101 MHz, C*D*$Cl_3$) $\delta$ 136.6, 127.7, 122.3, 122.1, 119.4, 119.3, 115.9, 111.2, 21.4.

IR (cm$^{-1}$): 3391 (NH).

Followed literature procedure, data matches literature data[22].

NMR Data:

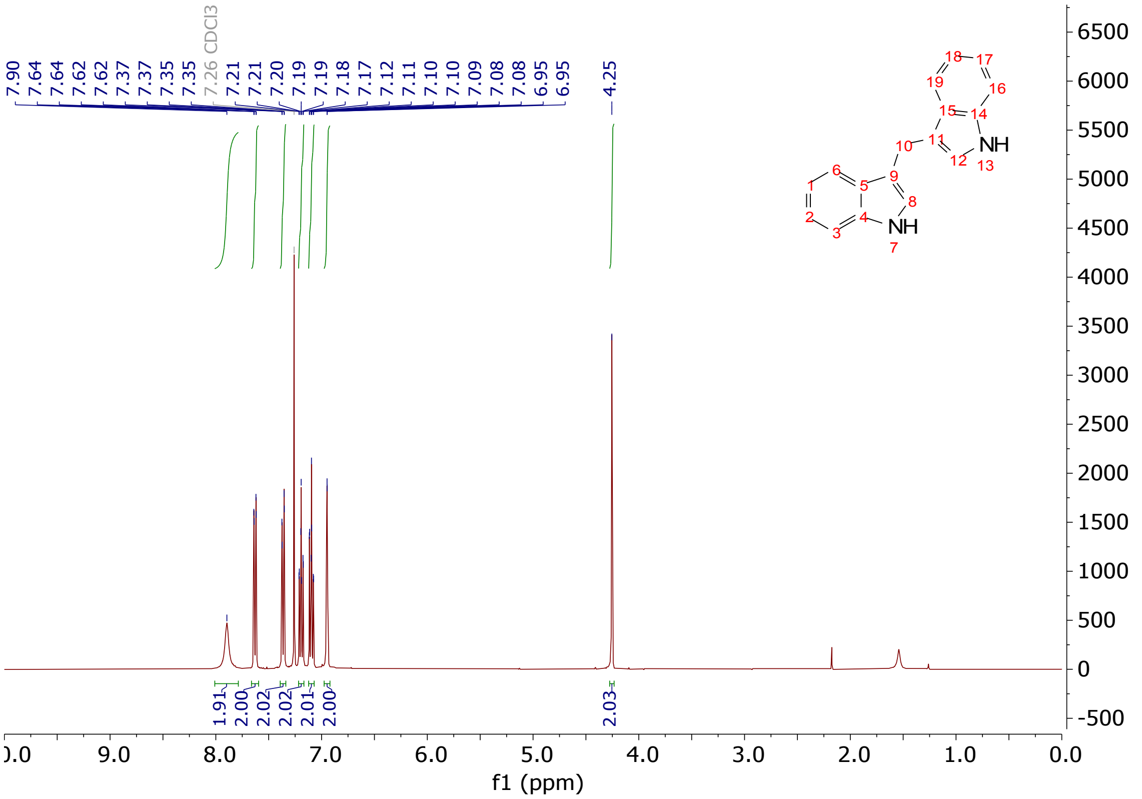


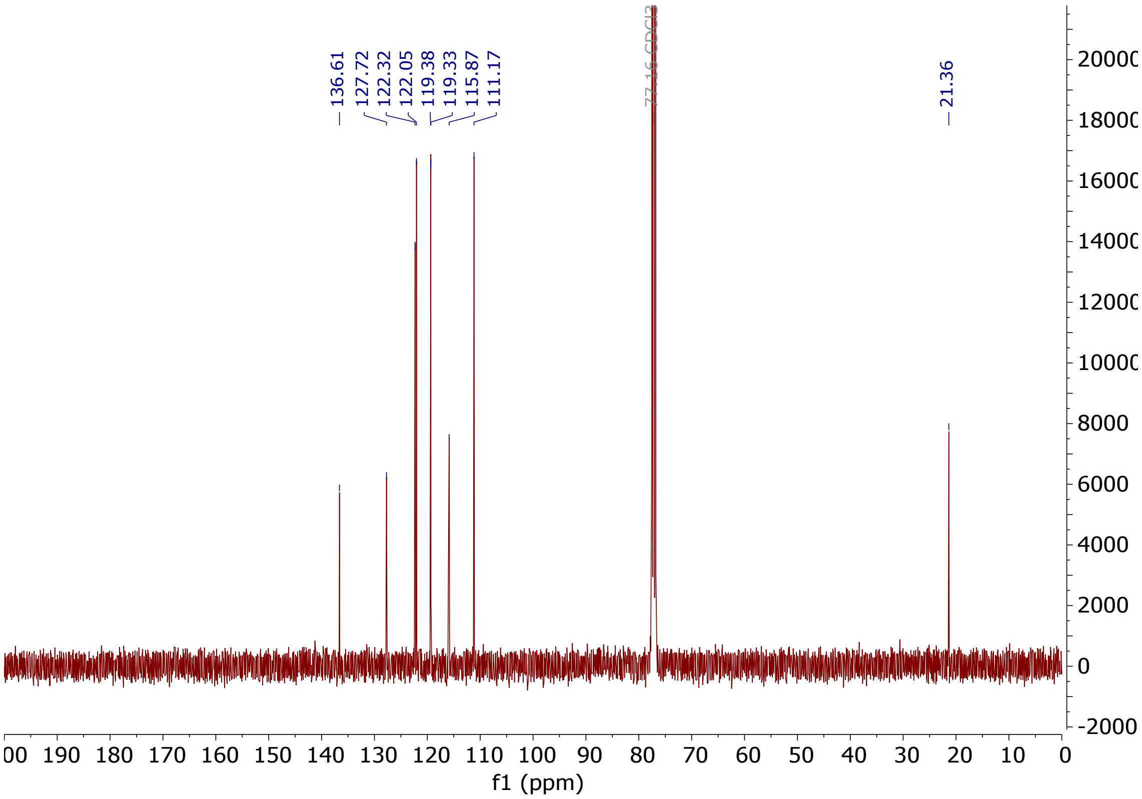


**Figure S19.** NMR data for **DIM** – 3,3'-diindolylmethane

## Additional References

The supporting information references 22 additional publications.


(1) Pichler, A.; Knipscheer, P.; Oberhofer, E.; Van Dijk, W. J.; Körner, R.; Olsen, J. V.; Jentsch, S.; Melchior, F.; Sixma, T. K. SUMO Modification of the Ubiquitin-Conjugating Enzyme E2-25K. *Nat Struct Mol Biol.* **2005**, *12*(3), 264-269, DOI: 10.1038/nsmb903.

(2) Murray, A. M.; Kelly, C. D.; Nussey, S. S.; Johnstone, A. P. Production of Glutathione-Coated Microtitre Plates for Capturing Recombinant Glutathione S-Transferase Fusion Proteins as Antigens in Immunoassays. *J Immunol Methods.* **1998**, *218*(1–2), 133-139, DOI: 10.1016/S0022-1759(98)00114-8.

(3) Watt, J. E.; Hughes, G. R.; Walpole, S.; Monaco, S.; Stephenson, G. R.; Bulman Page, P. C.; Hemmings, A. M.; Angulo, J.; Chantry, A. Discovery of Small Molecule WWP2 Ubiquitin Ligase Inhibitors. *Chem EurJ.* **2018**, *24*(67), 17677-17680, DOI: 10.1002/chem.201804169.

(4) Kathman, S. G.; Span, I.; Smith, A. T.; Xu, Z.; Zhan, J.; Rosenzweig, A. C.; Statsyuk, A. V. A Small Molecule That Switches a Ubiquitin Ligase from a Processive to a Distributive Enzymatic Mechanism. *J Am Chem Soc.* **2015**, *137*(39), 12442-12445, DOI: 10.1021/jacs.5b06839.

(5) Wang, Z.; Liu, Z.; Chen, X.; Li, J.; Yao, W.; Huang, S.; Gu, A.; Lei, Q. Y.; Mao, Y.; Wen, W. A Multi-Lock Inhibitory Mechanism for Fine-Tuning Enzyme Activities of the HECT Family E3 Ligases. *Nat Commun.* **2019**, *10*(1), 3162, DOI: 10.1038/s41467-019-11224-7.

(6) Madhavi Sastry, G.; Adzhigirey, M.; Day, T.; Annabhimoju, R.; Sherman, W. Protein and Ligand Preparation: Parameters, Protocols, and Influence on Virtual Screening Enrichments. *J Comput Aided Mol Des.* **2013**, *27*(3), 221-234, DOI: 10.1007/s10822-013-9644-8.

(7) Johnston, R. C.; Yao, K.; Kaplan, Z.; Chelliah, M.; Leswing, K.; Seekins, S.; Watts, S.; Calkins, D.; Chief Elk, J.; Jerome, S. V.; Repasky, M. P.; Shelley, J. C. Epik: PKa and Protonation State Prediction through Machine Learning. *J Chem Theory Comput.* **2023**, *19*(8), 2380-2388, DOI: 10.1021/acs.jctc.3c00044.

(8) Watts, K. S.; Dalal, P.; Tebben, A. J.; Cheney, D. L.; Shelley, J. C. Macrocycle Conformational Sampling with Macromodel. *J Chem Inf Model.* **2014**, *54*(10), 2680-2696, DOI: 10.1021/ci5001696.

(9) Mohamadi, F.; Richards, N. G. J.; Guida, W. C.; Liskamp, R.; Lipton, M.; Caufield, C.; Chang, G.; Hendrickson, T.; Still, W. C. Macromodel—an Integrated

Software System for Modeling Organic and Bioorganic Molecules Using Molecular Mechanics. *J Comput Chem.* **1990**, *11*(4), 440-467, DOI: 10.1002/jcc.540110405.

(10) Jorgensen, W.; Tirado-Rives, J. The OPLS [Optimized Potentials for Liquid Simulations] Potential Functions for Proteins, Energy Minimizations for Crystals of Cyclic Peptides and Crambin. *J Am Chem Soc.* **1988**, *110*(6), 1657-1666, DOI: 10.1021/ja00214a001.

(11) Jorgensen, W. L.; Maxwell, D. S.; Tirado-Rives, J. Development and Testing of the OPLS All-Atom Force Field on Conformational Energetics and Properties of Organic Liquids. *J Am Chem Soc.* **1996**, *118*(45), 2734-2742, DOI: 10.1021/ja9621760.

(12) Shivakumar, D.; Williams, J.; Wu, Y.; Damm, W.; Shelley, J.; Sherman, W. Prediction of Absolute Solvation Free Energies Using Molecular Dynamics Free Energy Perturbation and the Opls Force Field. *J Chem Theory Comput.* **2010**, *6*(5), 1509-1519, DOI: 10.1021/ct900587b.

(13) Harder, E.; Damm, W.; Maple, J.; Wu, C.; Reboul, M.; Xiang, J. Y.; Wang, L.; Lupyan, D.; Dahlgren, M. K.; Knight, J. L.; Kaus, J. W.; Cerutti, D. S.; Krilov, G.; Jorgensen, W. L.; Abel, R.; Friesner, R. A. OPLS3: A Force Field Providing Broad Coverage of Drug-like Small Molecules and Proteins. *J Chem Theory Comput.* **2016**, *12*(1), 281-296, DOI: 10.1021/acs.jctc.5b00864.

(14) Roos, K.; Wu, C.; Damm, W.; Reboul, M.; Stevenson, J. M.; Lu, C.; Dahlgren, M. K.; Mondal, S.; Chen, W.; Wang, L.; Abel, R.; Friesner, R. A.; Harder, E. D. OPLS3e: Extending Force Field Coverage for Drug-Like Small Molecules. *J Chem Theory Comput.* **2019**, *15*(3), 1863-1874, DOI: 10.1021/acs.jctc.8b01026.

(15) Halgren, T. A.; Murphy, R. B.; Friesner, R. A.; Beard, H. S.; Frye, L. L.; Pollard, W. T.; Banks, J. L. Glide: A New Approach for Rapid, Accurate Docking and Scoring. 2. Enrichment Factors in Database Screening. *J Med Chem.* **2004**, *47*(7), 1750-1759, DOI: 10.1021/jm030644s.

(16) Friesner, R. A.; Banks, J. L.; Murphy, R. B.; Halgren, T. A.; Klicic, J. J.; Mainz, D. T.; Repasky, M. P.; Knoll, E. H.; Shelley, M.; Perry, J. K.; Shaw, D. E.; Francis, P.; Shenkin, P. S. Glide: A New Approach for Rapid, Accurate Docking and Scoring. 1. Method and Assessment of Docking Accuracy. *J Med Chem.* **2004**, *47*(7), 1739-1749. DOI: 10.1021/jm0306430.

(17) Delano, W. L. The PyMOL Molecular Graphics System. *CCP4 Newsletter on protein crystallography*. **2002**, *40*(1), 44-53, pp 44-53.

(18) Kutschy, P.; Dzurilla, M.; Takasugi, M.; Török, M.; Achbergerová, I.; Homzová, R.; Rácová, M. New Syntheses of Indole Phytoalexins and Related Compounds. *Tetrahedron*. **1998**, *54*(14), 3549-3566, DOI: 10.1016/S0040-4020(98)00088-X.

(19) Wang, G.; Li, C.; He, L.; Lei, K.; Wang, F.; Pu, Y.; Yang, Z.; Cao, D.; Ma, L.; Chen, J.; Sang, Y.; Liang, X.; Xiang, M.; Peng, A.; Wei, Y.; Chen, L. Design, Synthesis and Biological Evaluation of a Series of Pyrano Chalcone Derivatives Containing Indole Moiety as Novel Anti-Tubulin Agents. *Bioorg Med Chem*. **2014**, *22*(7), 2060-2079, DOI: 10.1016/j.bmc.2014.02.028.

(20) Quirit, J. G.; Lavrenov, S. N.; Poindexter, K.; Xu, J.; Kyauk, C.; Durkin, K. A.; Aronchik, I.; Tomasiak, T.; Solomatin, Y. A.; Preobrazhenskaya, M. N.; Firestone, G. L. Indole-3-Carbinol (I3C) Analogues Are Potent Small Molecule Inhibitors of NEDD4-1 Ubiquitin Ligase Activity That Disrupt Proliferation of Human Melanoma Cells. *Biochem Pharmacol*. **2017**, *127*, 13-27, DOI: 10.1016/j.bcp.2016.12.007.

(21) Arcadi, A.; Calcaterra, A.; Chiarini, M.; Fabrizi, G.; Fochetti, A.; Goggiamani, A.; Iazzetti, A.; Marrone, F.; Marsicano, V.; Serraiocco, A. Synthesis of Indole/Benzofuran-Containing Diarylmethanes through Palladium-Catalyzed Reaction of Indolylmethyl or Benzofuranylmethyl Acetates with Boronic Acids. *Synthesis,* **2021**, *54*(3), 741-753. DOI: 10.1055/s-0041-1737275.

(22) Chao, W. R.; Yean, D.; Amin, K.; Green, C.; Jong, L. Computer-Aided Rational Drug Design: A Novel Agent (SR13668) Designed to Mimic the Unique Anticancer Mechanisms of Dietary Indole-3-Carbinol to Block Akt Signaling. *J Med Chem*. **2007**, *50*(15), 3412-3415, DOI: 10.1021/jm070040e.
